# CoQ10 targeted hippocampal ferroptosis in a status epilepticus rat model

**DOI:** 10.1007/s00441-024-03880-z

**Published:** 2024-03-19

**Authors:** Heba Fikry, Lobna A. Saleh, Faten A. Mahmoud, Sara Abdel Gawad, Hadwa Ali Abd-Alkhalek

**Affiliations:** 1 Department of Histology and Cell Biology, Faculty of Medicine, Ain Shams University, Khalifa El-Maamon st, Abbasiya sq., Cairo, 11566 Egypt; 2 Department of Clinical Pharmacology, Faculty of Medicine, Ain Shams University, Khalifa El-Maamon st, Abbasiya sq., Cairo, 11566 Egypt

**Keywords:** Status epilepticus, Coenzyme Q10, Ferroptosis, Oxidative stress, Lithium-pilocarpine, Rat

## Abstract

Status epilepticus (SE), the most severe form of epilepsy, leads to brain damage. Uncertainty persists about the mechanisms that lead to the pathophysiology of epilepsy and the death of neurons. Overloading of intracellular iron ions has recently been identified as the cause of a newly recognized form of controlled cell death called ferroptosis. Inhibiting ferroptosis has shown promise as a treatment for epilepsy, according to recent studies. So, the current study aimed to assess the possible antiepileptic impact of CoQ10 either alone or with the standard antiepileptic drug sodium valproate (SVP) and to evaluate the targeted effect of COQ10 on hippocampal oxidative stress and ferroptosis in a SE rat model. Using a lithium-pilocarpine rat model of epilepsy, we evaluated the effect of SVP, CoQ10, or both on seizure severity, histological, and immunohistochemical of the hippocampus. Furthermore, due to the essential role of oxidative stress and lipid peroxidation in inducing ferroptosis, we evaluated malonaldehyde (MDA), reduced glutathione (GSH), glutathione peroxidase 4 (GPX4), and ferritin in tissue homogenate. Our work illustrated that ferroptosis occurs in murine models of lithium-pilocarpine-induced seizures (epileptic group). Nissl staining revealed significant neurodegeneration. A significant increase in the number of astrocytes stained with an astrocyte-specific marker was observed in the hippocampus. Effective seizure relief can be achieved in the seizure model by administering CoQ10 alone compared to SVP. This was accomplished by lowering ferritin levels and increasing GPX4, reducing MDA, and increasing GSH in the hippocampus tissue homogenate. In addition, the benefits of SVP therapy for regulating iron stores, GPX4, and oxidative stress markers were amplified by incorporating CoQ10 as compared to SVP alone. It was concluded that CoQ10 alone has a more beneficial effect than SVP alone in restoring histological structures and has a targeted effect on hippocampal oxidative stress and ferroptosis. In addition, COQ10 could be useful as an adjuvant to SVP in protecting against oxidative damage and ferroptosis-related damage that result from epileptic seizures.

## Introduction

Recurrent generalized convulsions lasting more than 30 min constitute status epilepticus (SE), a common neurological disorder that, if left untreated, can cause irreversible neuronal damage in the brain. Nearly 30% of instances of progressive epilepsy are refractory, meaning they do not respond to pharmacological treatment (Dyomina et al. 2020; Josephson and Jetté 2017).

Iron is essential for healthy brain growth and function, and iron deficiency or iron overload can negatively affect the nervous system (Thirupathi and Chang 2019). There is a delicate balance of iron in the brain. Oligodendrocytes and astrocytes play an important role in maintaining that balance. Iron is also necessary for normal neurogenesis, myelination, and catecholamine neurotransmitter metabolism (Cheli et al. 2020). Neuroinflammation, neurodegeneration, and neurobehavioral impairments have all been linked to elevated iron levels in the brain, according to recent studies (Apostolakis and Kypraiou 2017; Cheli et al. 2020). In addition, recent research suggests that active epilepsy is an independent risk factor for the development and progression of COVID-19 (Kuroda 2021). These findings underline the importance of further safeguarding epileptic youth against contracting the COVID-19 virus.

Ferroptosis is a type of cell death that has only recently been recognized, and it is characterized by iron-dependent lipid peroxidation (Dixon et al. 2012; Li et al. 2018; Stockwell et al. 2017). It is characterized by the accumulation of intracellular iron ions, leading to the accumulation of lethal lipid-based reactive oxygen species (ROS) (Cai and Yang 2021). In ferroptosis, cellular signaling networks and genes control the buildup of iron-dependent free radicals and lipid oxidation products. Abnormal iron ion metabolism, depletion of reduced glutathione (GSH), glutathione peroxidase 4 (GPX4), and aberrant lipid peroxidation (malondialdehyde, MDA) are the three key components in ferroptosis. However, the precise regulatory network is unclear (Stockwell and Jiang 2020). Alzheimer’s disease, Parkinson’s disease, stroke, and traumatic brain injury are only some neurological disorders and illnesses linked to ferroptosis (Cho et al. 2020; Van Do et al. 2016). Although ferroptosis is known to have a part in the onset of seizures, its precise role in the genesis of seizures, particularly those triggered by pentylenetetrazol (PTZ) or pilocarpine (Pilo), is not well understood.

Moreover, oxidative stress is strongly linked to the induction of epileptic activity and the death of nerve cells and is caused by prolonged convulsions that encourage the excessive formation of ROS (Eastman et al. 2020; Freitas 2009), which end in lipid peroxidation and ferroptosis (Dixon et al. 2012; Wang et al. 2020a, b). In addition, many polyunsaturated fatty acids (PUFAs) in the membranes of neurons in the brain are vulnerable to lipid peroxidation. The brain is also rich in iron, which plays a role in producing hydroxyl radicals. In summary, when excessive iron ions are present in the cytoplasm, lipid peroxidation increases, producing harmful lipid free radicals and triggering ferroptosis. When more PUFAs are in the cells, lipid peroxidation increases, worsening ferroptosis (Cai and Yang 2021). These epileptic pathophysiological processes in the brain are related to ferroptosis because oxidative stress and lipid peroxidation are crucial in initiating ferroptosis (Lin et al. 2020). So, delaying the onset or severity of epilepsy may be aided by keeping ROS levels under tolerable control, which minimizes the incidence of ferroptosis.

Antiepileptic medications (AEDs) are currently the gold standard in epilepsy therapy. Unfortunately, about a third of all epilepsy patients did not respond to the most frequently prescribed AEDs, and these were the drug-refractory epilepsy patients (Moshé et al. 2015). One of the most often used antiepileptic medicines is sodium valproate (SVP) (Perucca 2002). There have been reports of both prooxidative and antioxidative effects of SVP on oxidative stress (Belcastro et al. 2013; Ezz et al. 2011). There is evidence that SVP therapy affects iron metabolism in epilepsy, leading to the production of non-transferrin-bound iron and an increase in OS (Ounjaijean et al. 2011). Therefore, new antiseizure therapies may also be developed by creating pharmaceuticals blocking the ferroptosis signaling axis.

Coenzyme Q10 (CoQ10) is a naturally occurring isoprenyl benzoquinone molecule that functions similarly to a vitamin and is created endogenously in the human body; that is synthesized in the inner mitochondrial membrane with a lipophilic character that makes it easy to diffuse through membranes (Garrido-Maraver et al. 2014a, b; Hernández-Camacho et al. 2018; Sifuentes-Franco et al. 2022). Meats, fish, salmon, sardines, pork, chicken, nuts, soybeans, vegetable oils, and many other foods contain CoQ10, albeit at much lower concentrations, with the remaining CoQ10 coming from biosynthesis within the body. Dairy products, fruits, and cereals also contain CoQ10, albeit at much lower concentrations (Pravst et al. 2010). CoQ10 has seen widespread use as a medicinal agent, with applications spanning from treating neurodegenerative disorders to those of heart failure, fibromyalgia, and even insulin resistance (Garrido-Maraver et al. 2014a, b; Pastor-Maldonado et al. 2020; Tawfik 2011). In addition, CoQ10 has gained popularity as a supplement for its potential to affect cellular bioenergetics and protect against free radical damage in recent years (Bhardwaj and Kumar 2016; Tawfik 2011).

Therefore, the current study aimed to assess the possible antiepileptic impact of CoQ10 either alone or with the standard antiepileptic drug sodium valproate (SVP) and to evaluate the targeted effect of COQ10 on hippocampal oxidative stress and ferroptosis in a SE rat model.

## Materials and methods

### Animals

From the Animal House of the Medical Research Center, Faculty of Medicine at Ain shams University, we received 6- to 8-week-old male adult Wistar rats weighing 200–250 g. The animals were housed in an institutional setting with a standard temperature regulation (22 3 °C) and a light/dark cycle of 12 h daily. Except during experiments, they had unrestricted access to food and drink. Within each group, there were ten animals.

### Ethical statement

All animal maintenance and procedures were established in agreement with institutional guidelines for animal care and use published by the National Institutes of health. In addition, the institutional Animals Care and Use Committee (ACUC) and Research Ethics Committee (FMASUS REC) approved the experimental protocol with Federal wide assurance No. 000175. 85 (Reference No. FMASU R222/2022).

### Chemicals and drugs

Lithium chloride (LiCl; Product Number: L4408), scopolamine methyl bromide (Product Number: S8502), pilocarpine hydrochloride (3S,4R)-3-ethyl-4-((1-methyl-1H-imidazol-5-yl) methyl) dihydrofuran-2(3H)-one (Product Number: PHR1493), and CoQ10 (C59H90O4; CAS Number: 303-98-0) diazepam (10 mg/kg) were purchased from Sigma-Aldrich, St. Louis, MO, USA. Sodium valproate (SVP) was obtained from a local pharmacy under the brand name Depakine. The ingredients (SVP, Pilo, and CoQ10) were dissolved in dimethyl sulfoxide (DMSO). The animals were weighed and received the calculated dose of the drug according to their weight. Enzyme-linked immunosorbent assay (ELISA) kits for reduced glutathione (GSH) (Catalog Number: MBS724319), lipid peroxidation (malondialdehyde, MDA) (Cat.: MBS9718963), glutathione peroxidase 4 (phospholipid hydroperoxides) (GPX4) (Cat.: MBS934198), and ferritin (FE) (Cat.: MBS2709273) were purchased from MyBioSource, Inc. San Diego, USA. Polyclonal antiferritin rabbit antibody (Cat.: MBS8247387, MyBioSource, Inc. San Diego, USA) and polyclonal primary anti GFAP goat antibody (Catalog # 13-0300, Thermo Scientific Co, Waltham, MA, USA) were used. Biotinylated secondary antibody (horse antimouse IgG antibodies (BA-2000-1.5), 1:500 diluted in PBS containing 0.05% Triton X‐100 and 2.5% horse serum) was obtained from Vector Laboratories, Burlingame, CA.

### Induction of SE by lithium-pilocarpine

To produce seizures, we utilized pilocarpine, diluted in DMSO (0.5 mg/ml), and injected intraperitoneally (i.p.) at 100 mg/kg dose. In brief, Pilo was administered i.p. to the Wistar rats every 20 min till the beginning of the limbic seizure. In most cases, seizures can be induced repeatedly with just three injections (Mao et al. 2019). Lithium chloride (LiCl; 127 mg/kg, i.p.) was administered 18 h before pilocarpine injection. Thirty minutes before the injection of Li-Pilo, scopolamine methyl bromide (1 mg/kg i.p) was administered to reduce any peripheral effects caused by pilocarpine (Davis 2013; Juvale and Has 2020). Diazepam (10 mg/kg, i.p) was given 75 min after the onset of score four seizures to stop SE and reduce mortality associated with prolonged seizure activity (Fan et al. 2020).

### Experimental design

**Fig. 1:**
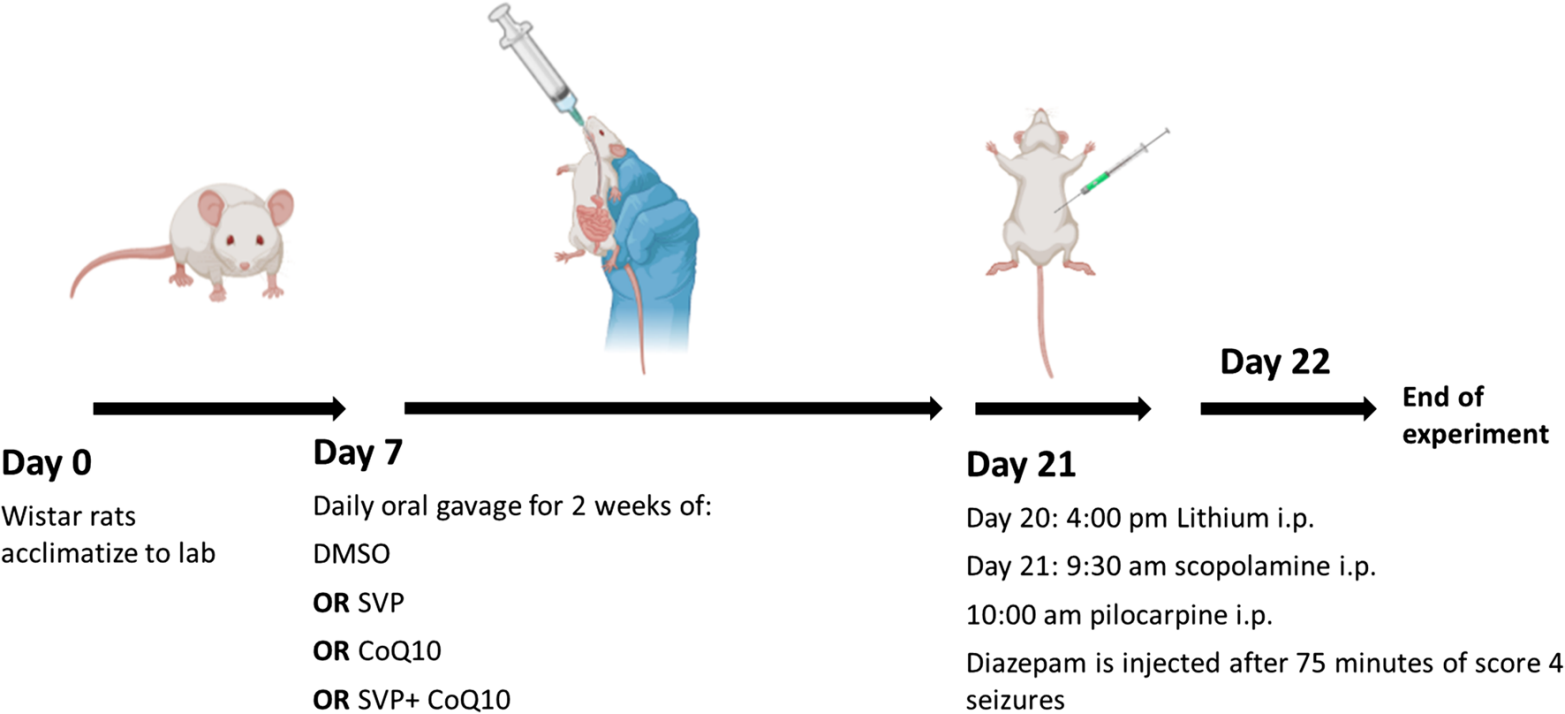
Experimental design

Fifty Wistar Albino rats were randomly allocated to 5 groups, ten animals each, as follows (Fig. 1):

- **Control I (vehicle group):** Rats received DMSO by gavage daily for 2 weeks and were injected with lithium, scopolamine methyl bromide, and diazepam, like the Pilo-treated group, except for three injections of DMSO instead of Pilo.
- **Group II (epileptic group):** Before receiving an injection of Pilo, rats were given DMSO via gavage once a day for 2 weeks.
- **Group III (SVP-treated group):** SVP (300 mg/kg body weight) was given by gavage daily for 2 weeks before Pilo injection from the start of the study (Rashid et al. 2021).
- **Group IV (Coq10-treated group):** From the beginning of the study, rats were given CoQ10 (20 mg/kg) via gavage once a day for 2 weeks before they got a Pilo injection (Tawfik 2011).
- **Group V (SVP + Coq10-treated group):** At the outset of the trial, rats were given SVP + Coq10 via gavage once daily for 2 weeks before they were injected with Pilo.

### Assessment of epileptogenesis

Animals were observed for 1 h after Pilo administration for the onset and severity of seizures. Rat seizure intensity was evaluated by observing the animals’ actions. Initial akinesia, whole-body tremor, and/or incomplete limbic gustatory automatisms, salivation, and head-scratching were all logged as indicators of impending convulsive activity. Seizure onset latency and maximum seizure duration (in minutes) were calculated. The severity of seizures was rated using the Racine scale (Racine 1972): 1 = seizure consisted of immobility and occasional facial clonus; 2 = head nodding; 3 = bilateral forelimb clonus; 4 = rearing; 5 = rearing and falling. According to the criteria established by prior research, SE was considered to have occurred when there were three or more unprovoked seizures (Modebadze et al. 2016).

All rats were injected i.p with 1.2 gm/kg urethane 24 h after the experiment was finished to induce anesthesia. Urethane is advantageous because it provides a continuous, long-lasting anesthetic without interfering with neurotransmission in the brain’s subcortical regions or periphery. Moreover, urethane-anesthetized animals have physiological and pharmacological behaviors comparable to non-anesthetized animals (Maggi and Meli 1986). First, the rats were perfused transcardially with ice-cold physiological saline. Then, ice-cold physiological saline was infused transcardially into the rats. The brain was successfully removed through a posterior neck incision. The brain was exposed after the skull was carefully removed. Both the left and right cerebral hemispheres were removed from the brain. The hippocampi from the left-brain halves were removed immediately and frozen at − 80 °C for later biochemical analysis. The right hemispheres were preserved in 10% neutral buffered formalin for histological and immunohistochemical analysis.

### Biochemical study

For biochemical determinations, the hippocampus tissue samples were minced and homogenized separately in ice-cold PBS (0.02 mol/l, pH 7.0–7.2) at a concentration of 15% (w/v). After two freeze–thaw cycles were performed to break the cell membranes, the homogenate was centrifuged at 130,000 × g for 10 min at 2–8 °C. Remove the supernate and assay immediately or aliquot and store samples at − 20 °C or − 80 °C (Rabuffetti et al. 2000).

#### Detection of oxidative stress parameters

These parameters include malondialdehyde (MDA) as an index for lipid peroxidation and antioxidant enzymes as reduced glutathione (GSH). Tissue homogenate MDA levels were calculated using the Ohkawa et al. method of reacting the sample with thiobarbituric acid (Ohkawa et al. 1979). The spectrophotometric detection for MDA at optical density (O.D.) 532 and 600 nm. The MDA result was given in terms of nmol/mg of protein. Elliott’s method for measuring GSH concentration was used (Ellman 1959). The microplate reader’s spectrophotometer is set to read at 450 nm to determine the color’s intensity. GSH levels were measured, and the result was reported in ng/mg of protein.

#### Detection of ferritin (FE) accumulation in hippocampus tissue

The test principle applied in this kit is sandwich enzyme immunoassay according to the manufacturer’s protocols. This kit includes a pre-coated microtiter plate immunostained with an antibody against FE. After that, a biotin-conjugated antibody specific to FE is added to the wells of the relevant microtiter plate. As a next step, avidin-conjugated to conjugated horseradish peroxidase (HRP) is applied to each microplate well and incubated. Only the wells that originally contained FE, biotin-conjugated antibody, and enzyme-conjugated avidin will show a color shift. The color change is determined spectrophotometrically at a wavelength of 450 nm ± 10 nm after a sulphuric acid solution has stopped the enzyme-substrate reaction. Next, the FE concentration in the samples is calculated by comparing their optical densities to the mean curve. The FE result was reported in ng/mg of protein.

#### Detection of glutathione peroxidase 4 (phospholipid hydroperoxides) (GPX4)

The sandwich enzyme immunoassay method is used for quantification in accordance with the manufacturer’s instructions. A microplate has been pre-coated with an antibody against GPX4. Each well contains an immobilized antibody specific for GPX4, which binds to GPX4 in pipetted samples. As soon as the wells are free of unattached compounds, a biotin-conjugated antibody directed against GPX4 is added. After the wells have been cleaned, avidin-conjugated HRP is added. After the wells have been washed to eliminate any unbound avidin-enzyme reagent, a substrate solution is added, and color develops in response to the initial GPX4 binding concentration. A stop in the development of the color is made, and the degree of that color is recorded. Color saturation is determined by spectrophotometric analysis in a microplate reader by illuminating the sample at 450 nm. The GPX4 result was given in terms of pg/mg protein.

#### Protein assay

Bradford assay was used to determine the protein concentration in the supernatant, and bovine serum albumin (BSA, Sigma Chemical, USA) was used as the reference standard (Bradford 1976).

### Histological evaluation of hematoxylin and eosin (H&E) and Nissl staining

After being fixed in 10% neutral buffered formalin, coronal sections of the right hemisphere were washed, dehydrated, cleaned in xylol, and embedded in paraffin. For histological analysis of the rat hippocampus, paraffin-embedded tissue slides were stained with H&E for and Nissl stain to observe neuronal loss according to Bancroft and Layton (Suvarna et al. 2019).

### Immunohistochemical study

Different immunocytochemical markers were stained on horizontal sections of both control and treatment animals. Two levels of analysis were performed for each animal. For 30 min, sections were incubated in a solution of 0.3 percent hydrogen peroxide in phosphate-buffered saline (PBS) at pH 7.4 to deactivate endogenous peroxidase after being rinsed in 0.05 M PBS at pH 7.4. After two 10-min washes in PBS, the sections were rinsed for 60 min in PBS plus 0.4% BSA. A polyclonal antiferritin rabbit antibody was used to incubate a single hippocampal section from each group. Ferritin does not label neurons, whereas microglia and oligodendrocytes are (de Rodríguez‐Callejas et al. 2019). Another section of the hippocampus of all groups was incubated with primary polyclonal antiGFAP goat antibody. Astrocytes’ location and response to brain degeneration or damage are most commonly studied with GFAP (Martin and O’Callaghan 1995). The avidin–biotin immunoperoxidase method with modifications was used. Working dilution was 1:1000 in PBS for 1 h for 30 min at room temperature. Sections were washed in PBS, incubated with the secondary antibody for an hour at room temperature, and then washed again. Streptavidin peroxidase was applied at room temperature for 10 min before being rinsed with PBS. The reactions were visualized with 3′, 3 regular diaminobenzidine tetrahydrochloride (DAB). The sections were counterstained with Mayer’s hematoxylin, dehydrated, and mounted.

Their brown cell membranes and cytoplasm identified immunoreactive oligodendrocytes and microglia for ferritin and astrocytes with GFAP. Negative control sections were successfully produced by following the same protocol but substituting PBS for the primary antibody.

### Morphometric study

The morphometrics of all groups’ specimens was analyzed. Captured at × 40 magnification were analyzed using the Leica Q win V. 3 program installed on a computer in the Histology and Cell Biology Department, Faculty of Medicine, Ain Shams University. An attached Leica DM2500 microscope (Wetzlar, Germany) was used in conjunction with the computer. Histologists performing the morphometric analyses were blinded to the pathologic diagnoses obtained by examining the specimens from different groups. Five slides were taken from each specimen and used for measurements. For each slide, we measured the following in five non-overlapping fields of CA1, CA3, and DG in the hippocampus:

1. Vaibhav et al. methods were used to estimate neuron loss. First, dead cells were assumed to have pyknotic nuclei. Next, the percentages of viable and non-viable cells in each sample were determined. Finally, neuronal loss was defined as the ratio of dead cells to total viable cells (H&E-stained sections) (Vaibhav et al. 2013).
2. The positively stained cells with a well-defined nucleolus and typical Nissl bodies were counted (Nissl-stained sections) (Wang et al. 2020a, b).
3. The number of ferritin-positive cells (de Rodríguez‐Callejas et al. 2019).
4. The number of GFAP-positive cells (GFAP immunostained sections) (Zhang et al. 2015).

### Statistical analysis

The Statistical Package for the Social Sciences (SPSS) for WINDOWS, version 26; IBM Corp., Armonk/New York, USA) was used to analyze the data. The data were displayed as the mean ± standard error of the mean (SEM) from all the groups. The Kolmogorov–Smirnov and Shapiro–Wilk tests were used to examine the normality of distribution. Analysis of variance (ANOVA) was used to see the differences between the groups, and then we used the LSD multiple comparisons test to dig further into the data. A *p*-value of less than 0.05 is considered statistically significant. The Pearson correlation test studied the association of GPX4 and biochemical variables. To create the graphs, we utilized GraphPad Prism Statistical Package for Windows, Version 9.3.1 (2021), San Diego, CA, USA.

## Results

### Detection and evaluation of signs of seizure activity and seizure severity

**Fig. 2:**
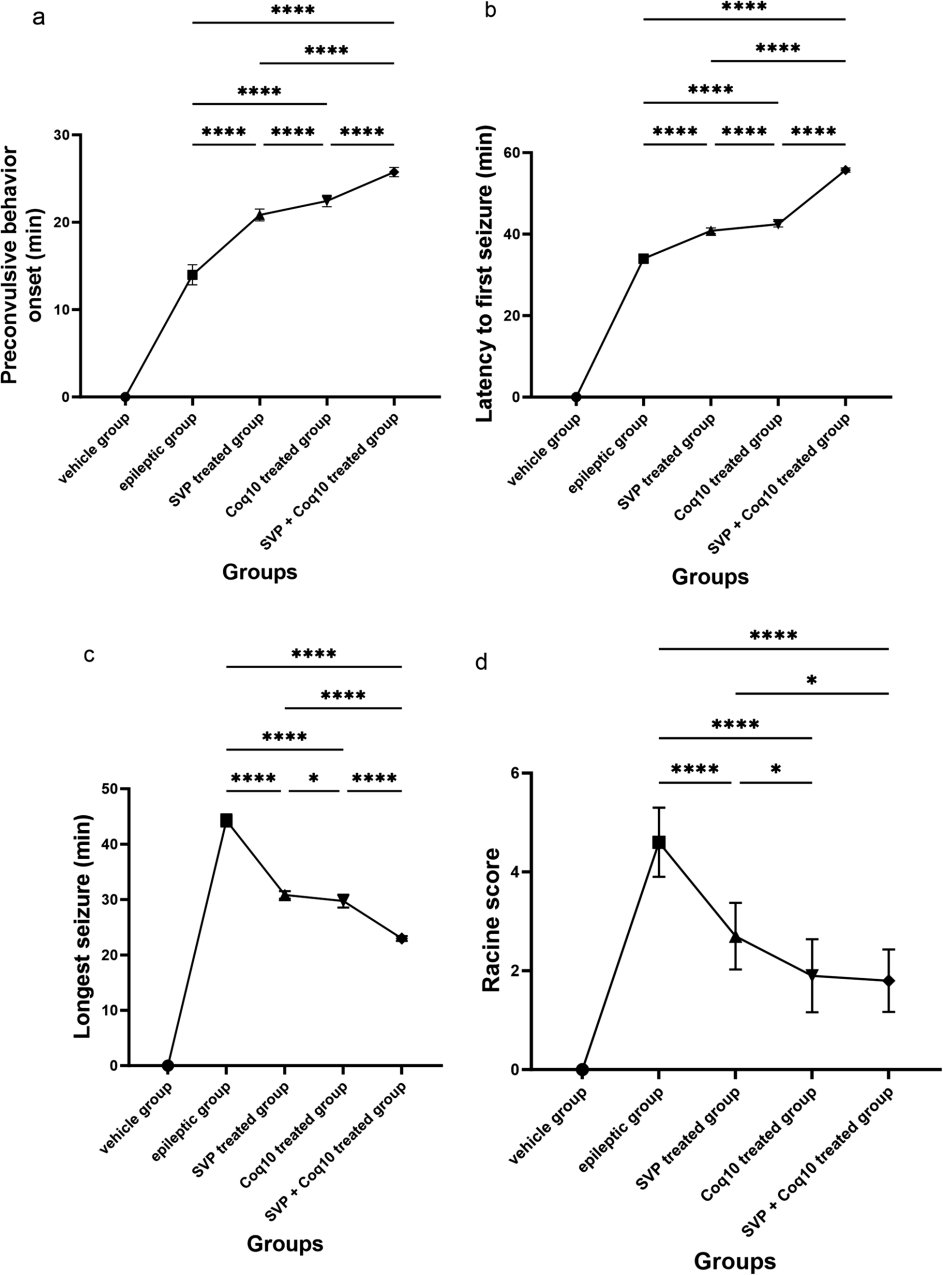
Effect of COQ10 either alone or in combination with sodium valproate on pilocarpine-induced seizures. **a** Preconvulsive behavior onset (min), **b** latency to first seizure (min), **c** longest seizure (min), and **d** Racine score. Significance at *p < 0.05, ****p < 0.0001

The effect of pilocarpine to induce preconvulsive behavior was seen in 13.99 ± 0.36 min. In the wake of these alterations, rats began experiencing attacks of continuous-stage seizures, which are associated with limbic motor seizures. The epileptic group showed the average Racine score was 4.60 ± 0.22, and the average delay to onset was 34.00 ± 0.36 min. The longest motor seizure typically lasted 44.29 ± 0.35 min. The latency to preconvulsant behavior was significantly (*P* < 0.0001) increased in SVP and CoQ10-pretreated rats (40.84 ± 0.21, 42.44 ± 0.20, respectively), and the latency to clonic and tonic seizures was significantly (*P* < 0.0001) increased in both groups compared to the epileptic group. Compared to the epileptic group, the rats’ significant (*P* < 0.0001) average Racine score decreased from 2.70 ± 0.21 to 1.90 ± 0.23, and the duration of their seizures decreased from 30.84 ± 0.21 to 29.74 ± 0.36 min. However, CoQ10 alone (1.90 ± 0.23) or in combination with SVP (1.80 ± 0.20) resulted in a significantly (*P* = 0.033, *P* = 0.0122, respectively) lower Racine score in CoQ10 alone compared to rats receiving SVP alone. The anticonvulsant effects of SVP were enhanced by 2 weeks of CoQ10 pretreatment. In rats, there was a statistically significant (*P* < 0.0001) delay in the onset of preconvulsant activity (Fig. 2a–d).

### Biochemical results

#### Lipid peroxidation and antioxidant enzyme activity

**Fig. 3:**
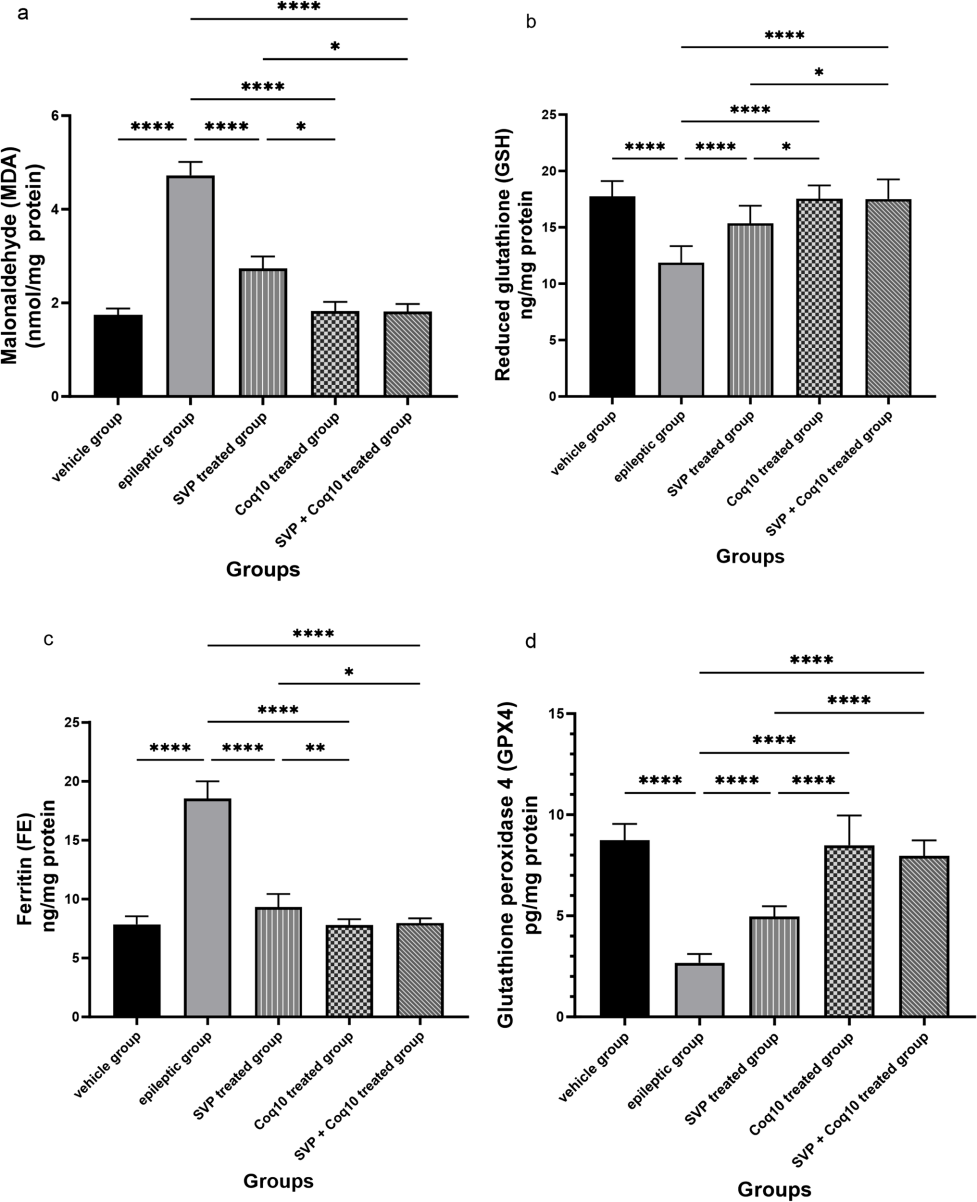
Effect of COQ10 either alone or in combination with sodium valproate on **a** malondialdehyde (MDA) as an index for lipid peroxidation, **b** antioxidant enzymes as reduced glutathione (GSH), **c** glutathione peroxidase 4 (GPX4), and **d** ferritin (FE) in hippocampus tissue. Significance at *p < 0.05, **p < 0.01, ****p < 0.0001

Figure 3a demonstrates that compared to the control group (1.74 ± 0.13), the SVP group, the CoQ10 group, and the SVP + CoQ10 group, the MDA levels in the epileptic model rat group were significantly higher (4.72 ± 0.28, *P* < 0.0001). MDA levels in rats treated with CoQ10 (1.82 ± 0.19 vs. 2.73 ± 0.80 in rats treated with SVP) were significantly (*P* = 0.04) lower. The group receiving SVP + CoQ10 (1.82 ± 0.15) showed a significantly decreased MDA than in the epileptic group (*P* = 0.03) and SVP-only group (*P* < 0.0001). In addition, CoQ10 and SVP + CoQ10 groups showed a non-significant difference (*P* > 0.99, *P* > 0.99, respectively) versus the control group.

Moreover, in Fig. 3b, the epileptic model rat group exhibited a significant (*P* < 0.0001) reduction in the **GSH** (11.86 ± 0.46) as compared to control, SVP, CoQ10, and SVP + CoQ10-treated groups. CoQ10 (17.55 ± 0.37) exhibited a significant (*P* = 0.017) increase in **GSH** in contrast with rats receiving SVP (15.36 ± 0.49). The group receiving the combination of both drugs (SVP + CoQ10; 17.52 ± 0.54) showed a significantly (*P* < 0.0001, *P* = 0.0196, respectively) increased **GSH** in contrast with an epileptic group and SVP-treated rats. Moreover, CoQ10 and SVP + CoQ10 groups exhibited non-significant differences (*P* > 0.99, *P* > 0.99, respectively) versus the control group (17.74 ± 0.43).

#### Detection of ferritin (FE) accumulation in hippocampus tissue

As shown in Fig. 3c, lithium-pilocarpine caused a significant (*P* < 0.0001) increase in the **FE** (18.53 ± 0.46) as compared to control, SVP, CoQ10, and SVP + CoQ10-treated groups. CoQ10 (7.79 ± 0.15) resulted in a significant (*P* = 0.006) decrease in **FE** in contrast with rats receiving SVP (9.31 ± 0.35). The group receiving the combination of both drugs (SVP + CoQ10; 7.96 ± 0.12) exhibited a significantly (*P* = 0.02, *P* < 0.0001, respectively) decreased **FE** than in the epileptic group and SVP-treated rats. Moreover, CoQ10 and SVP + CoQ10-treated groups showed non-significant differences (*P* > 0.99, *P* > 0.99, respectively) as compared to the control group (7.85 ± 0.21).

#### Detection of glutathione peroxidase 4 (phospholipid hydroperoxides) (GPX4)

**Fig. 4:**
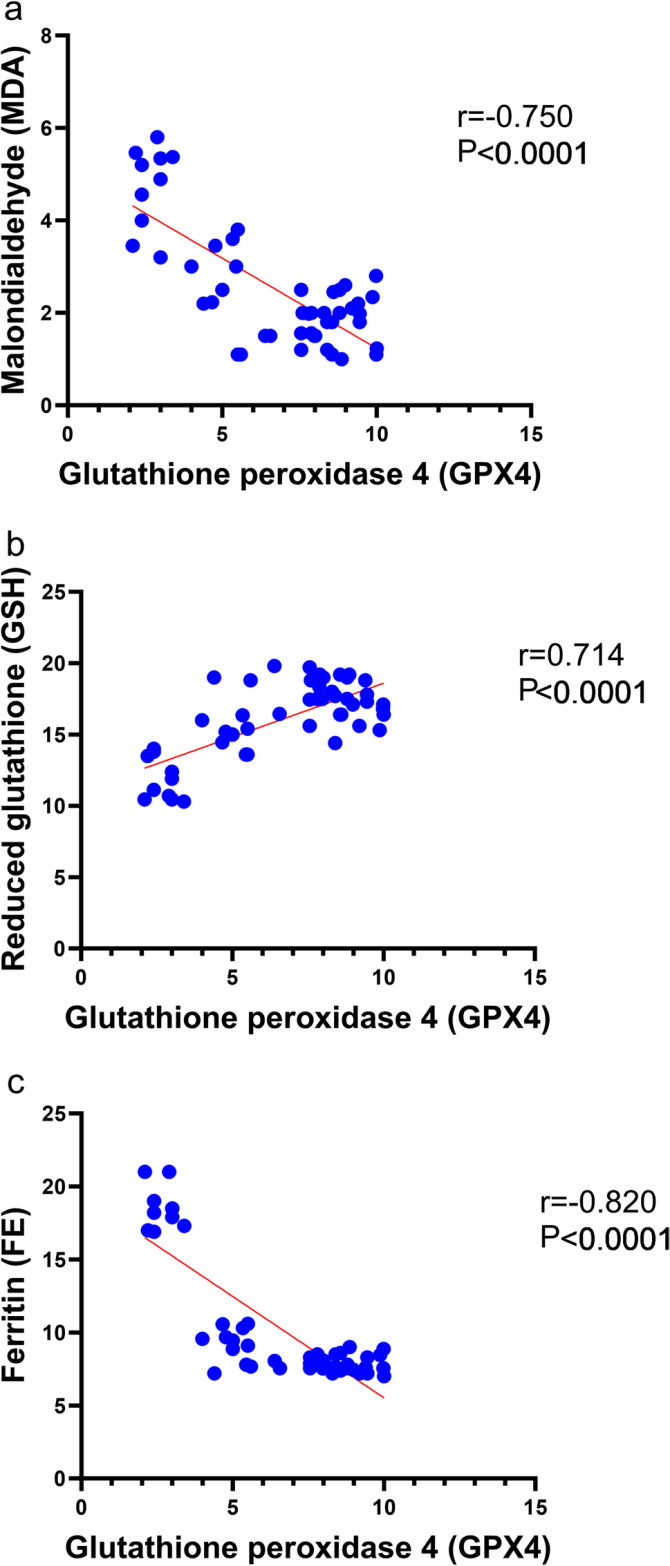
Correlation analysis of glutathione peroxidase 4 (GPX4) and biochemical parameters in hippocampus tissue (Pearson’s correlation, *n* = 50). Data expressed as **a** malondialdehyde (MDA) as an index for lipid peroxidation, **b** antioxidant enzymes as reduced glutathione (GSH), and **c** ferritin (FE)

As shown in Fig. 3d, lithium-pilocarpine caused a significant (*P* < 0.0001) reduction in the **GPX4** (2.68 ± 0.13) as compared to control, SVP, CoQ10, and SVP + CoQ10-treated groups. CoQ10 (8.48 ± 0.46) resulted in a significant (*P* < 0.0001) increase in **GPX4** in contrast with rats receiving SVP (4.96 ± 0.16). The group receiving the combination of both drugs (SVP + CoQ10; 7.97 ± 0.24) showed a significantly (*P* = 0.02, *P* < 0.0001, respectively) increased **GPX4** in contrast with an epileptic group and SVP-treated rats. CoQ10 and SVP + CoQ10-treated groups showed non-significant differences (*P* > 0.99, *P* = 0.45, respectively) versus the control group (8.73 ± 0.25). In addition, multiple significant associations were found when we compared the measured GPX4 and biochemical parameters across all the groups using Pearson’s correlation coefficient. There was a significant negative correlation in **GPX4** with **MDA** (*r* =  − 0.752, *P* < 0.0001) and **FE** (*r* =  − 0.820, *P* < 0.0001). While there was a significant positive correlation between **GPX4** and **GSH** (*r* = 0.714, *P* < 0.0001) **(**Fig. 4a–c**)**.

### Histological results

#### H&E

The H&E-stained sections of the hippocampus revealed two distinct regions: the dentate gyrus and the hippocampus proper. Cornu ammonis (CA) is made up of the hippocampal proper’s polymorphic layer, pyramidal layer, and molecular layer. As a dark V-shaped structure, the dentate gyrus (DG) can be seen in the images. The DG is a structure that comprises molecular, granule cell, and polymorphic layers.

##### Results of CA1

**Fig. 5:**
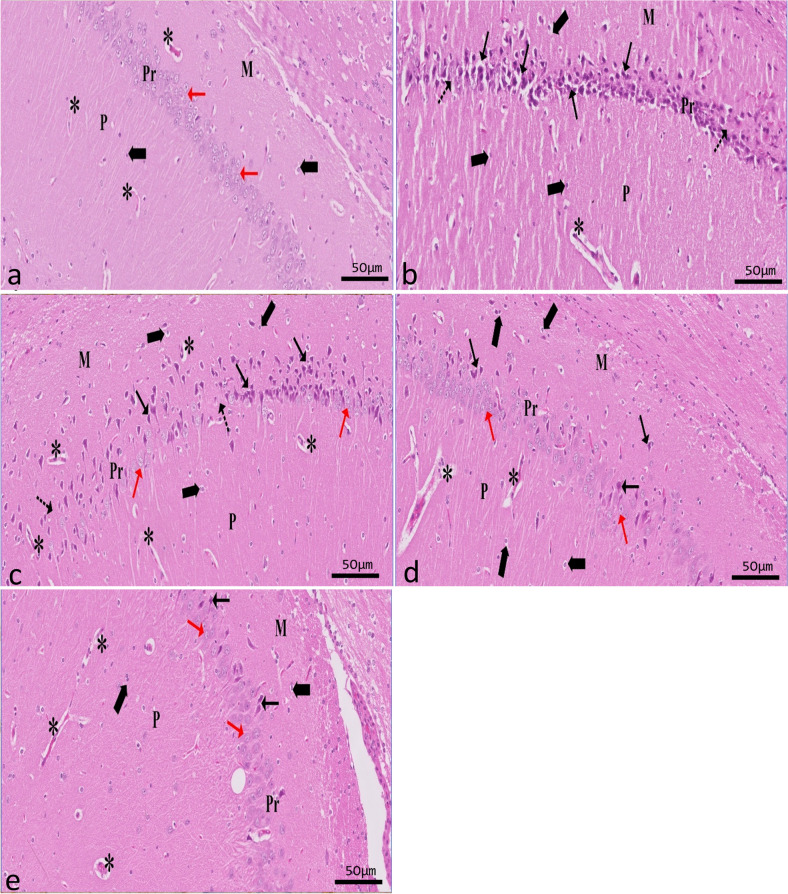
A sagittal section in rat hippocampus showing CA1 in different experimental groups (H&E ×20, scale bar 50 μm). Control group (**a**) shows that its pyramidal (Pr) layer is made up of five or six tightly packed layers of tiny pyramidal neurons (red↑) with vesicular nuclei. Both the molecular (M) layer and polymorphic (P) layer show neuroglial cells (thick arrow) with dark basophilic stained small, rounded nuclei, and perinuclear halos among neuropil (*) and small blood vessels. Epileptic group (**b**) shows degenerated pyramidal cells with deeply stained elongated nuclei with pericellular haloes (↑). Note the absence of pyramidal cells in a few areas (dot arrow). Both M and P layers contain congested blood capillaries among wide neuropils (*). An apparent increase of neuroglial cells (thick arrow). SVP-treated group (**c**) shows mild improvement, and some pyramidal cells appear degenerated (↑) among normal pyramidal cells (red↑). An apparent mild decrease of glial cells (thick arrow). M and P layers contain few congested blood capillaries among wide neuropils (*). CoQ10-treated group (**d**) and SVP + CoQ10-treated group (**e**) showing the layers were comparable to the control group. Most of the large pyramidal cells have vesicular nuclei (red↑). In addition, few degenerated cells with pericellular vacuolation (↑) are noticed. Glial cell interneurons are seen nearly as a control group in all layers of the hippocampus (thick arrow)

Analysis of the control vehicle group’s CA1 region showed that its pyramidal layer is made up of five or six tightly packed layers of tiny pyramidal neurons with vesicular nuclei. The molecular and polymorphic layers showed neuroglial cells with small dark basophilic rounded nuclei and perinuclear halos among neuropil and small blood vessels (Fig. 5a). Results suggested a thinner pyramidal layer in the CA1 region of the epileptic group. Some regions showed complete loss of pyramidal cells, while others showed degeneration marked by shrunken, deeply stained elongated nuclei or pyknotic nuclei with pericellular vacuolation. Degenerated neurons in the molecular and polymorphic layers were surrounded by perineurial glial cells. Blood capillaries were visibly congested in the molecular and polymorphic layers, which were otherwise characterized by wide neuropils (Fig. 5b). The CA1 region of the hippocampus showed modest improvement in the SVP-treated group compared to the epileptic group. Most pyramidal cells were normal, and only a small percentage of them showed deeply stained pyknotic nuclei with pericellular vacuolation. Pyramidal cells were found to be losing out in some regions. Few dilated capillaries were found among the vast neuropils of the molecular and polymorphic layers (Fig. 5c). Comparatively, the CoQ10-treated group was similar to the control group, although a subset of pyramidal cells had a reduced number of intensely pigmented, elongated nuclei and pericellular vacuolation. However, only a few swollen blood vessels were visible amid the neuropils. Neuroglial cells appeared to be decreasing in the molecular and polymorphic layers (Fig. 5d). However, the pyramidal cells in the SVP + CoQ10-treated group appeared almost similar to those in the control group, with only a few degenerated cells. On top of that, the molecular and polymorphic layers showed typical neuroglia (Fig. 5e).

##### Results of CA3

**Fig. 6:**
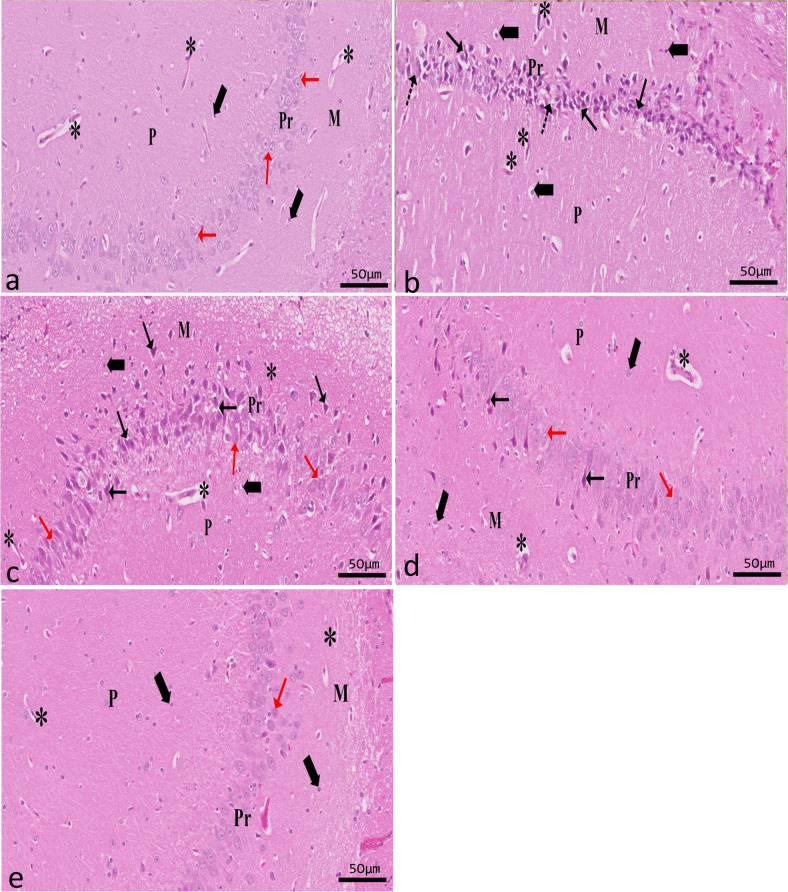
A sagittal section in rat hippocampus showing CA3 in different experimental groups (H&E ×20, scale bar 50 μm). The control group (**a**) shows numerous large pyramidal (red↑) neurons with vesicular nuclei seen in the pyramidal layer. Neuroglial cells (thick arrow) and small blood vessels are scattered among an eosinophilic background of the neuropil (*). The pyramidal layer of the epileptic group (**b**) appears severely disorganized, with degenerated pyramidal cells with deeply stained elongated nuclei and pericellular haloes (↑). Note the absence of pyramidal cells in a few areas (dot arrow). Molecular (M) and polymorphic (P) layers contain congested blood capillaries among wide neuropils (*). An apparent increase of neuroglial cells (thick arrow). The SVP-treated group (**c**) exhibited mild improvement compared to the epileptic group. Some pyramidal cells appeared degenerated (↑), with pericellular vacuolation among normal pyramidal cells (red↑). Both molecular and polymorphic layers show small blood capillaries among neuropils (*). Few pyramidal cells with deeply stained nuclei and pericellular vacuolation (↑) appear among regularly arranged pyramidal cells (red↑) in CoQ10-treated group (**d**). Neuroglia (thick arrow) and small blood vessels are scattered among the wide neuropil (*). SVP + CoQ10-treated group (**e**) appears nearly similar to the control group

Numerous large pyramidal neurons with vesicular nuclei were seen in the CA3 pyramidal layer of the control vehicle group. Neuroglial cells and small blood vessels were scattered among an eosinophilic background of the neuropil (Fig. 6a). The pyramidal layer in the epileptic group appeared to be severely disorganized, with several areas of cell death. Most of the pyramidal cells were shrunken, darkly pigmented, and surrounded by pericellular haloes. The molecular and polymorphic layers showed congested blood capillaries among wide neuropils. An apparent increase in the number of neuroglial cells among all layers was seen (Fig. 6b). The SVP-treated group exhibited mild improvement in all layers of the CA3 region as compared to the epileptic group. Some shrunken, deeply stained pyramidal cells with pericellular vacuolation were seen among normal pyramidal cells with vesicular nuclei. The molecular and polymorphic layers showed few congested blood capillaries among wide neuropils (Fig. 6c). Few pyramidal cells with deeply elongated nuclei and pericellular vacuolation were seen in the pyramidal layer in the CoQ10-treated group, but otherwise, there were no significant differences with the control group. However, few congested blood capillaries appeared among neuropils. Notice an apparent decrease of the glial cells could be observed scattered in the molecular and polymorphic layers (Fig. 6d). The SVP + CoQ10-treated group showed the same results as the control group. Most pyramidal cells appeared scattered with vesicular nuclei relatively similar to the control. Also, the molecular and polymorphic layers looked normal (Fig. 6e).

##### Results of DG

**Fig. 7:**
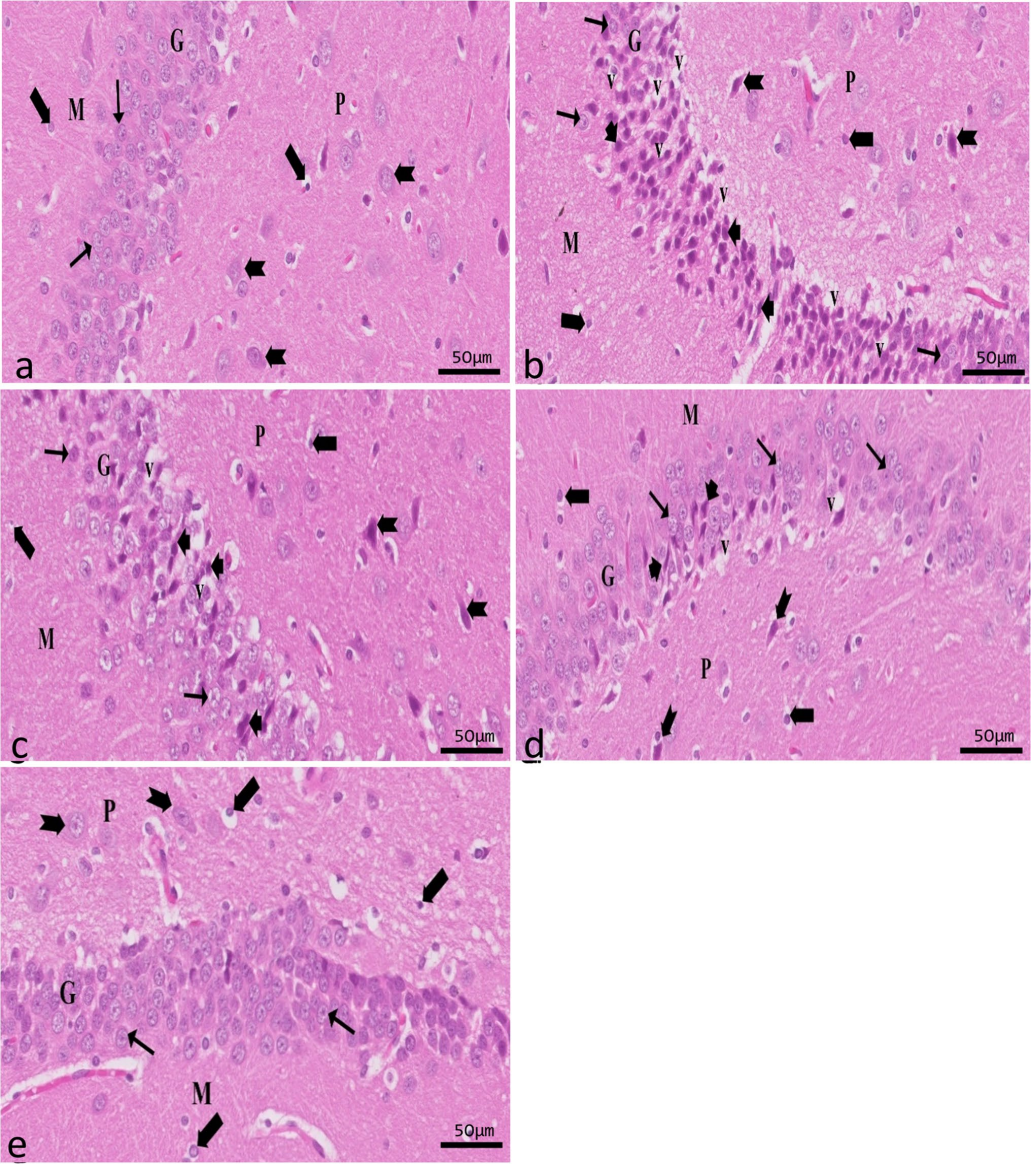
A sagittal section in rat hippocampus showing DG in different experimental groups (H&E ×20, scale bar 50 μm). Control group (**a**) showing the granular (G) layer is formed of a dense aggregate of granular cells that appeared rounded with vesicular nuclei (↑) with little interstitial tissue in-between. The polymorphic (P) layer contains scattered large pyramidal cells (bifid arrow) with vesicular nuclei, while neuroglial cells (thick arrow) appear scattered in both the molecular layer (M) and polymorphic layer (P). An epileptic group (**b**) shows many degenerated granule cells (arrowhead) are seen with a massive area of vacuolation (V). In addition to dark-stained apoptotic large pyramidal cells (bifid arrow) in the polymorphic layer (P), there is an apparent increase in the neuroglial cells (thick arrow). SVP-treated group (**c**) shows mild improvement. Few normal granular cells (↑) appeared among degenerated cells (arrowhead) with pericellular vacuolation (V). Both M and P layers show normal glial cells (thick arrow) darkly stained pyramidal cells (bifid arrow) with pericellular halos are found in the polymorphic layer (P). CoQ10-treated group shows few degenerated granular cells with pericellular vacuolation (V) (**d**). SVP + CoQ10-treated group shows similar results to the control group (**e**)

The granular layer of the control group was a compact sheet of granular cells with spherical nuclei. While neuroglial cells make up the molecular layer, the polymorphic layer has a few pyramidal cells with vesicular nuclei (Fig. 7a). Compared to the control group, the epileptic group’s DG showed degenerative alterations. Most of the granular cells appeared degenerated with pericellular vacuolation. Pyramidal cells of the polymorphic layer showed darkly stained pyknotic nuclei. Also, an apparent increase in neuroglial cells was seen (Fig. 7b). The SVP-treated group exhibited mild improvement in DG cell layers compared to the epileptic group. Few normal granular cells appeared among degenerated cells with pericellular vacuolation (Fig. 7c). A small number of granular cells with pericellular haloes were observed in the CoQ10-treated group; otherwise, there were no significant differences compared to the control (Fig. 7d). SVP and CoQ10-treated group appeared similar to the control group (Fig. 7e).

### Immunohistochemical (IHC) results

**Fig. 8:**
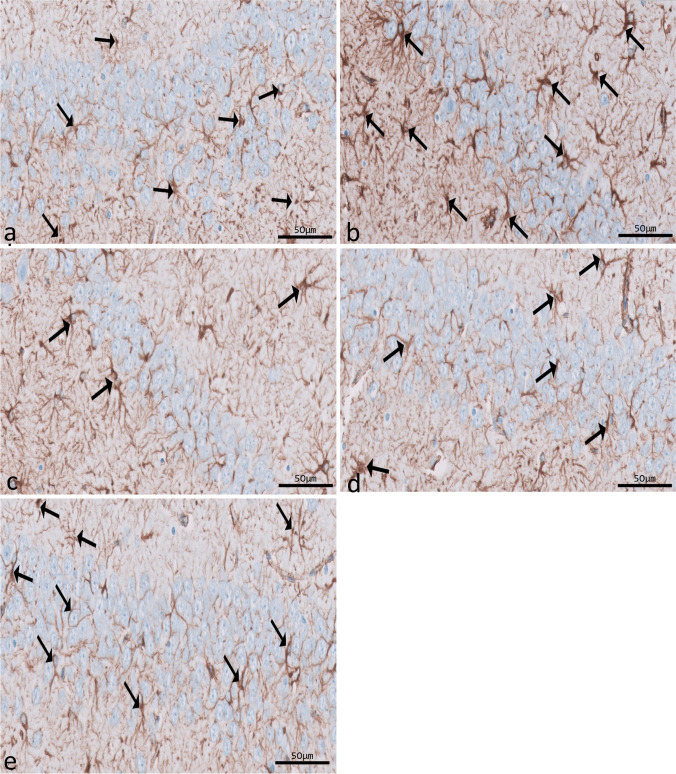
Immunohistochemical reaction for ferritin in sections of CA1 area of the hippocampus in different groups: control group (**a**), epileptic group (**b**), SVP-treated group (**c**), CoQ10-treated group (**d**), and SVP + CoQ10-treated group (**e**). Black arrows (↑) indicate positive immunohistochemical ferritin expression cells (×40, scale bar 50 μm)

**Fig. 9:**
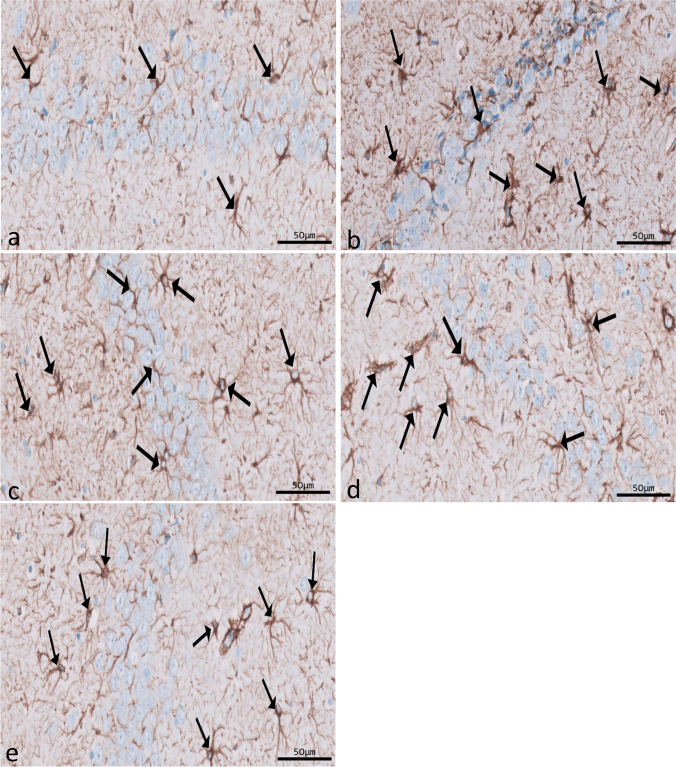
Immunohistochemical reaction for ferritin in sections of CA3 area of the hippocampus in different groups: control group (**a**), epileptic group (**b**), SVP-treated group (**c**), CoQ10-treated group (**d**), and SVP + CoQ10-treated group (**e**). Black arrows (↑) indicate positive immunohistochemical ferritin expression cells (×40, scale bar 50 μm)

**Fig. 10:**
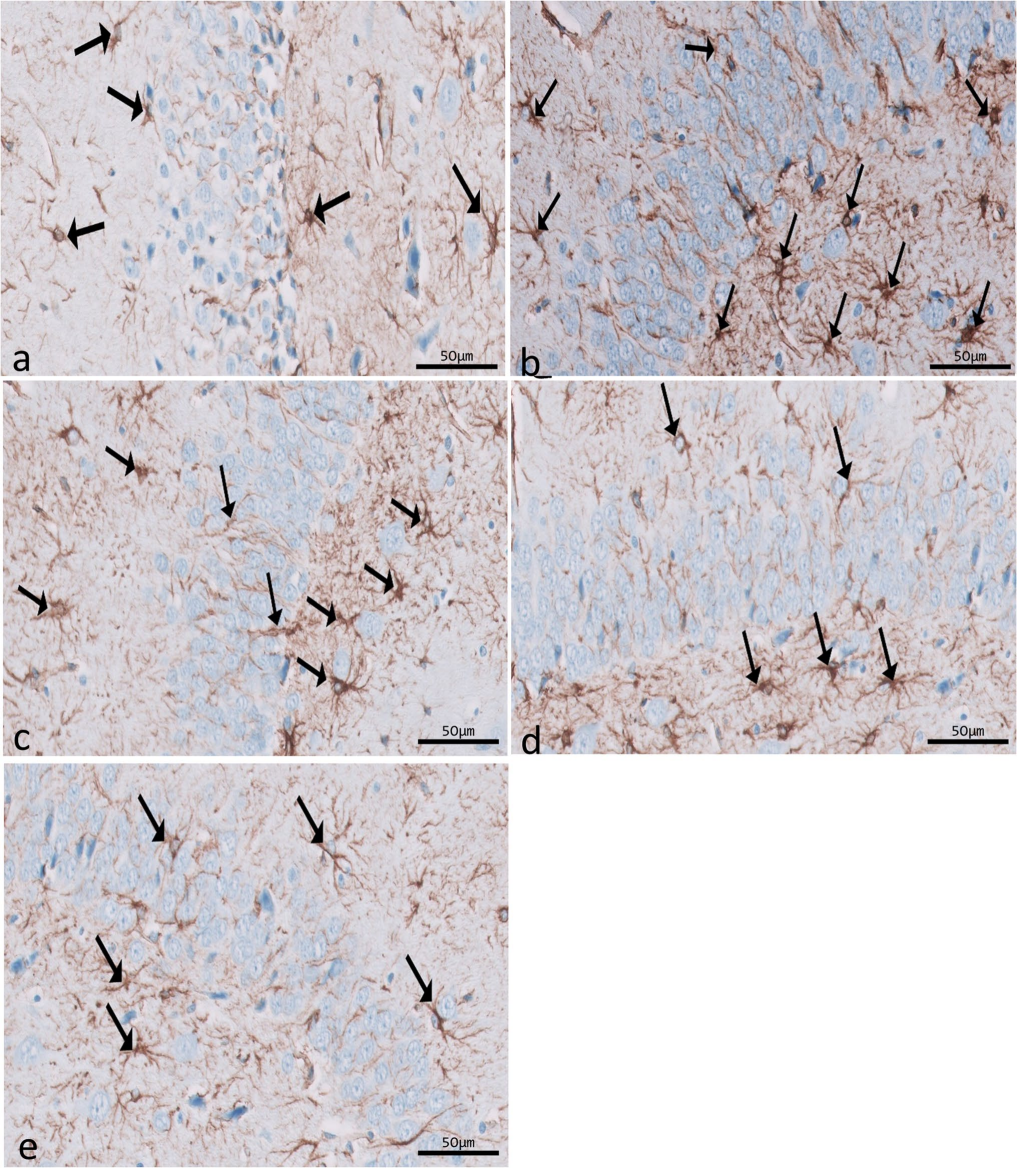
Immunohistochemical reaction for ferritin in sections of dentate gyrus (DG) in different groups: control group (**a**), epileptic group (**b**), SVP-treated group (**c**), CoQ10-treated group (**d**), and SVP + CoQ10-treated group (**e**). Black arrows (↑) indicate positive immunohistochemical ferritin expression cells (×40, scale bar 50 μm)

**Fig. 11:**
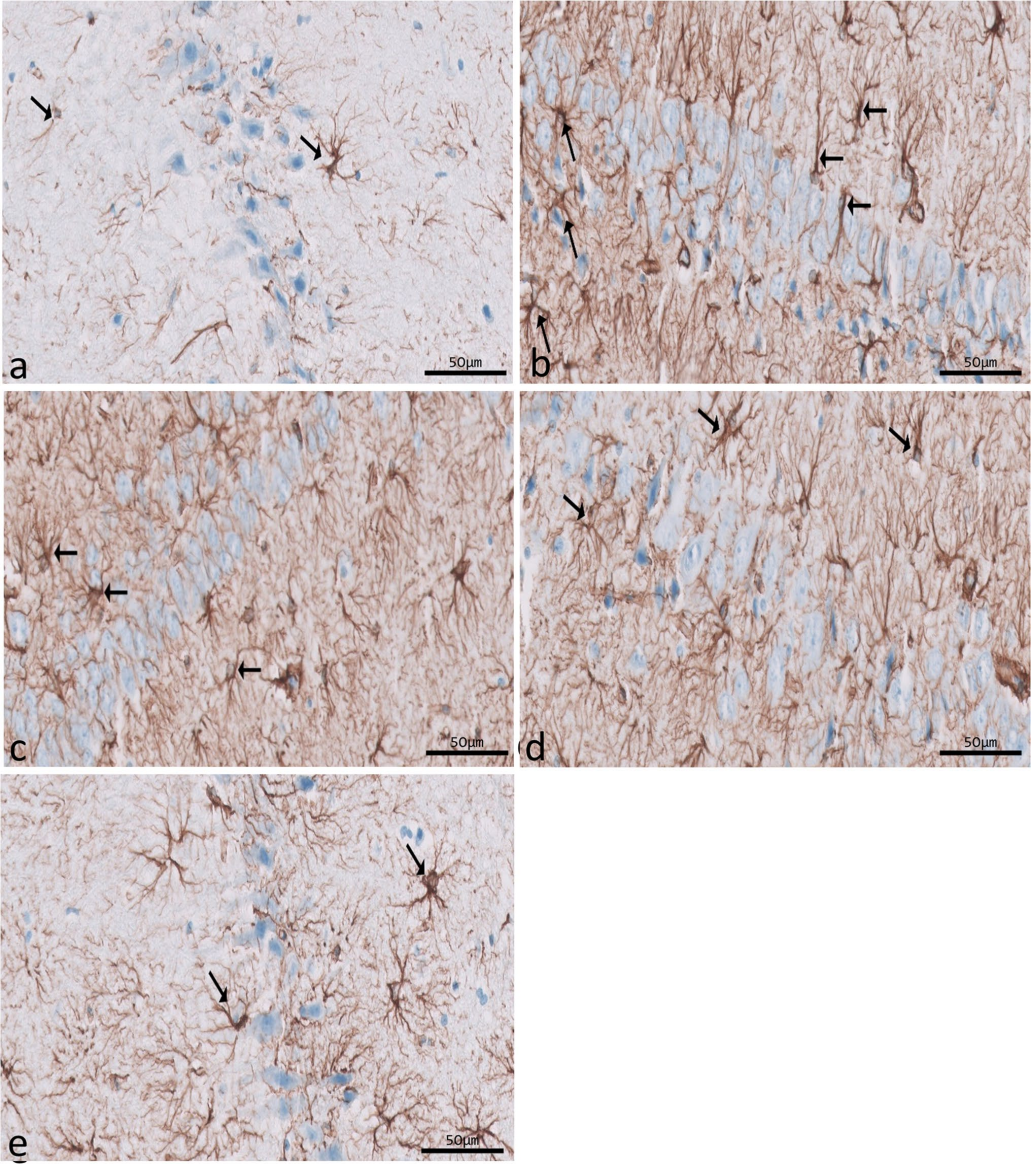
Immunohistochemical reaction for GFAP in sections of CA1 (**a**–**e**) area of the hippocampus in different groups. Control group (**a**), epileptic group (**b**), SVP-treated group (**c**), CoQ10-treated group (**d**), and SVP + CoQ10-treated group (**e**). Black arrows (↑) indicate positive cytoplasmic reactions in the body and processes of astrocytes (×40, scale bar 50 μm)

**Fig. 12:**
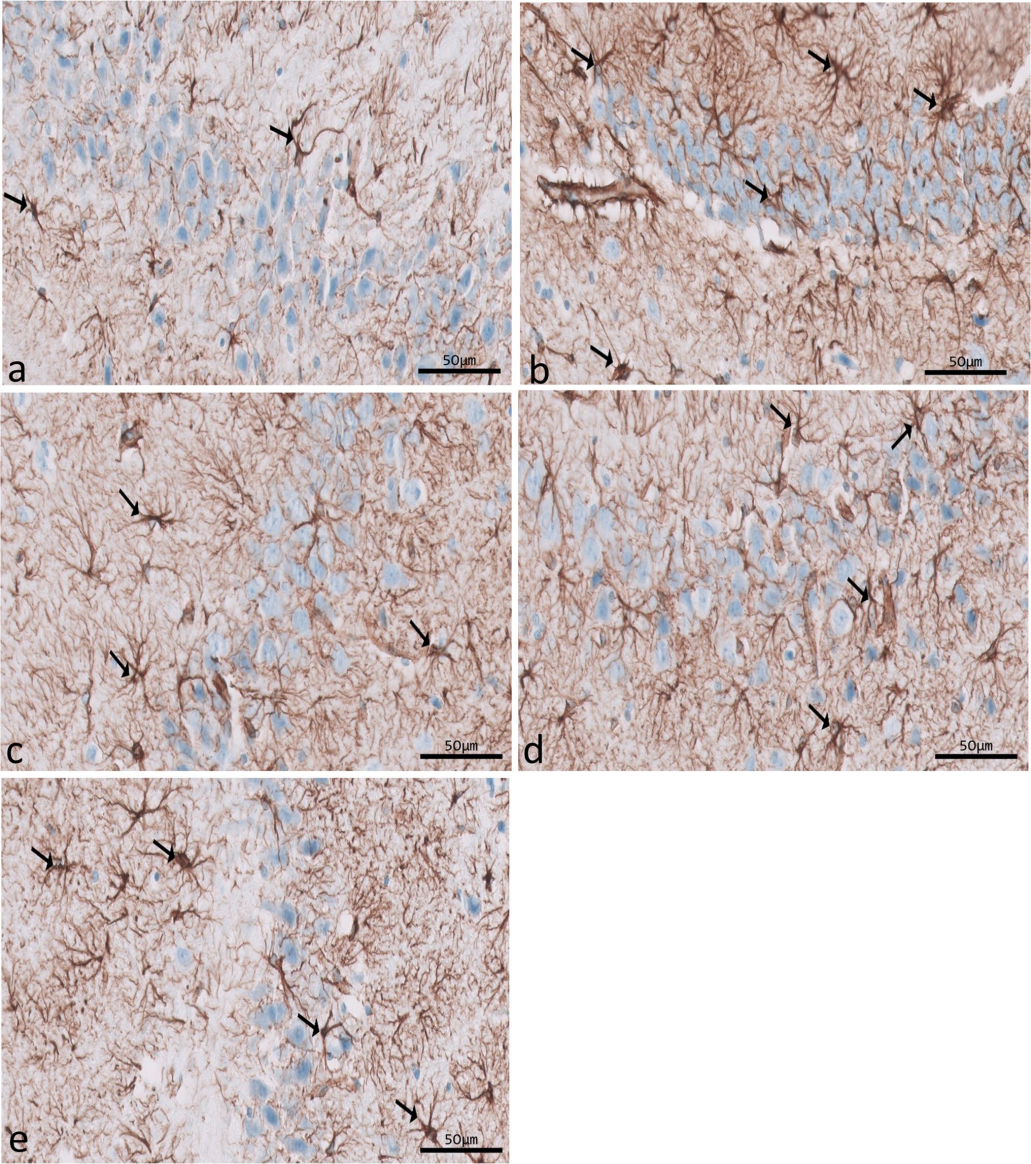
Immunohistochemical reaction for GFAP in sections of CA3 area of the hippocampus in different groups. Control group (**a**), epileptic group (**b**), SVP-treated group (**c**), CoQ10-treated group (**d**), and SVP + CoQ10-treated group (**e**). Black arrows (↑) indicate positive cytoplasmic reactions in the body and processes of astrocytes (×40, scale bar 50 μm)

**Fig. 13:**
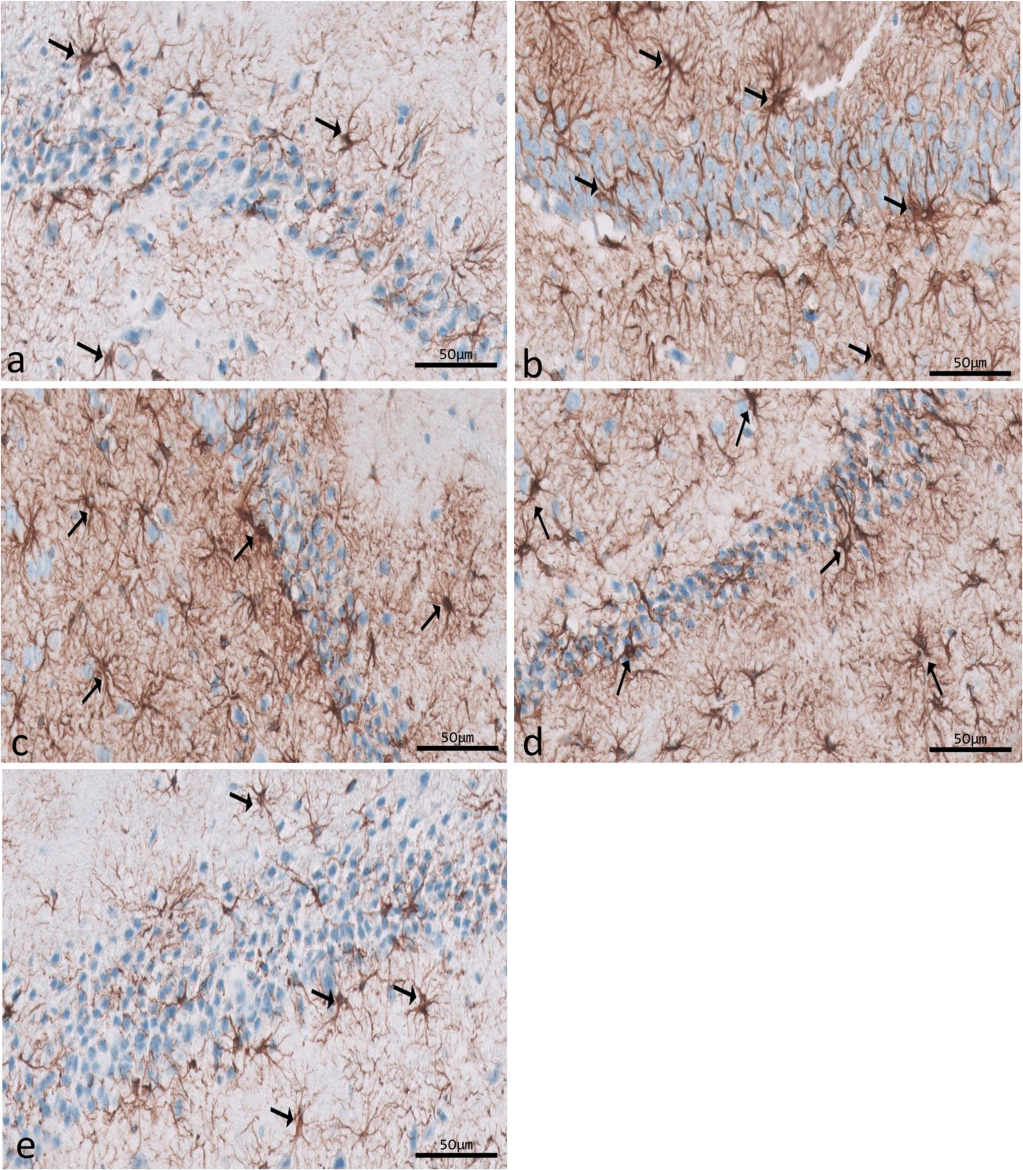
Immunohistochemical reaction for GFAP in sections of dentate gyrus (DG) in different groups. Control group (**a**), epileptic group (**b**), SVP-treated group (**c**), CoQ10-treated group (**d**), and SVP + CoQ10-treated group (**e**). Black arrows (↑) indicate positive cytoplasmic reactions in the body and processes of astrocytes (×40, scale bar 50 μm)

Immunohistochemical reactions for ferritin in sections of CA1 (Fig. 8a–e) and CA3 (Fig. 9a–e) areas and DG (Fig. 10a–e) were seen in different experimental groups. Hippocampal sections of the control group showed diffuse immunostaining for a small number of brownish neuropils in CA1, CA3, and DG areas. The epileptic group showed an apparent increase in ferritin-immunostained cells in the three areas. The SVP group revealed less increase in ferritin immunostained cells. Ferritin immunostained sections of coQ10-treated and SVP + coQ10-treated groups showed results nearly as that of the control group. Immunohistochemical reactions for GFAP in sections of CA1 (Fig. 11a–e) and CA3 (Fig. 12a–e) areas and DG (Fig. 13a–e) were seen in different experimental groups. The control group revealed a few GFAP-positive immunoreactive astrocytes dispersed in CA1, CA3, and DG. The epileptic group showed an apparent increase in GFAP immunoreactive astrocytes. The SVP group revealed moderately strong positive cytoplasmic reactions in most astrocytes compared to the epileptic group. Interestingly, GFAP-stained sections for the CoQ10-treated group and SVP + CoQ10-treated group showed results similar to the control.

### Morphometric results

**Fig. 14:**
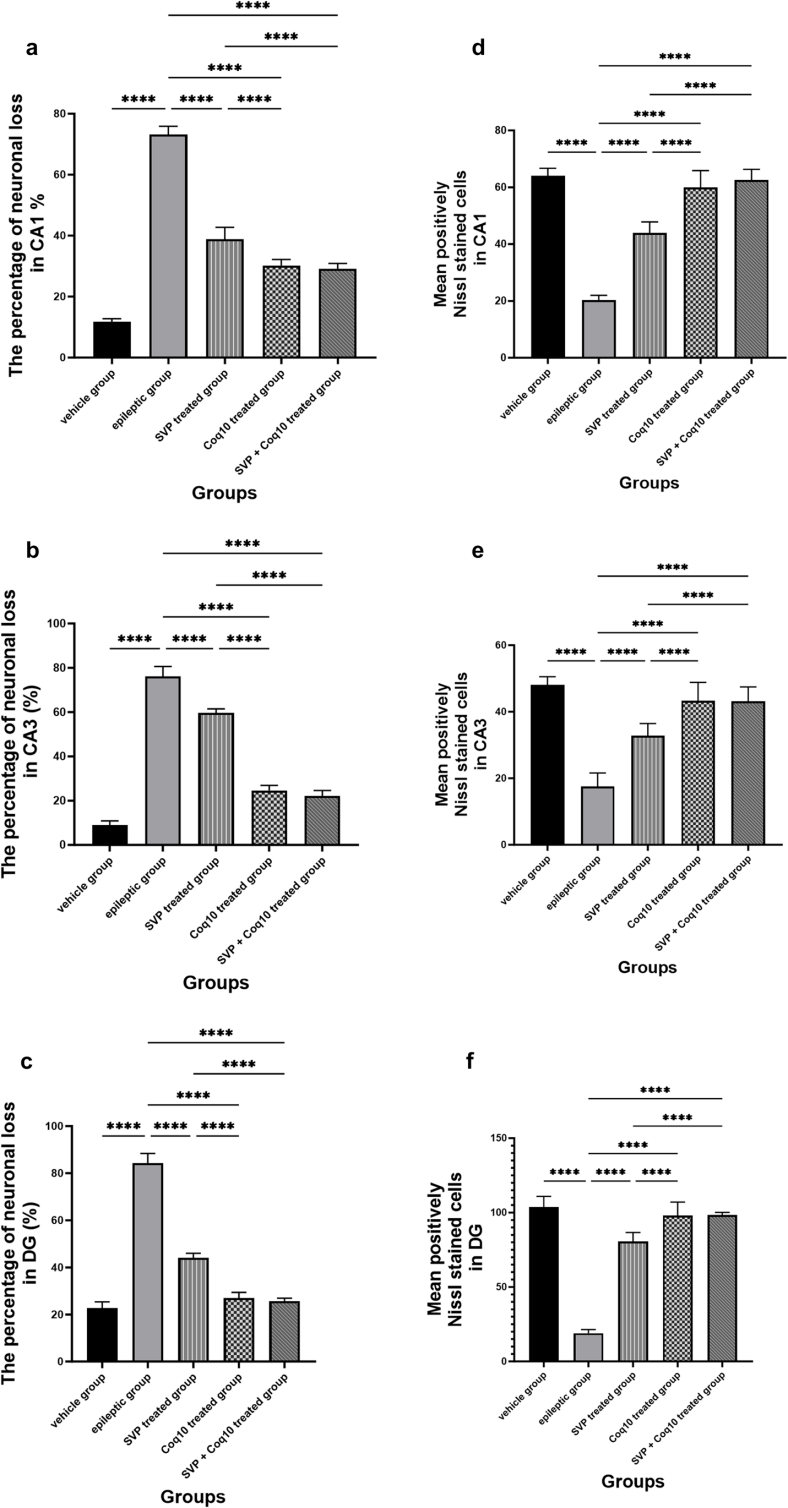
Effect of COQ10 alone or combined with sodium valproate on the morphometric hippocampus results of the hippocampus in different groups in CA1, CA3, and DG regions. The percentage of neuronal cell loss (**a**–**c**) and the percentage of positive Nissl-stained cells (**d**–**f**). ****Significant at *p* < 0.0001

As shown in Fig. 14a–c, lithium-pilocarpine caused a significant (*P* < 0.0001) increase in the percentage of neuronal cell loss in CA1 (73.18 ± 2.70), CA3 (76.17 ± 1.42), and DG (84.33 ± 1.28) as compared to control, SVP, CoQ10, and SVP + CoQ10-treated groups. CoQ10 resulted in a significant (*P* = 0.0001) decrease in the percentage of neuronal cell lose in CA1 (30.22 ± 0.62), CA3 (24.62 ± 0.72), and DG (27.02 ± 0.75) in contrast with rats receiving SVP in CA1 (38.83 ± 1.23), CA3 (59.62 ± 0.57), and DG (44.14 ± 0.59).

The group receiving the combination of both drugs (SVP + CoQ10) showed a significant (*P* < 0.0001) decrease in the percentage of neuronal cell loss in CA1 (29.14 ± 0.55), CA3 (22.10 ± 0.78), and DG (25.70 ± 0.39) than in an epileptic group and SVP-treated rats. In addition, SVP + CoQ10-treated groups showed a non-significant difference (*P* > 0.98, *P* > 0.39, *P* > 0.95, respectively) versus the CoQ10 group in CA1, CA3, and DG.

As shown in Fig. 14d–f, lithium-pilocarpine caused a significant (*P* < 0.0001) decrease in the percentage of positive Nissl-stained cells in CA1 (20.30 ± 0.53), CA3 (17.50 ± 1.29), and DG (18.90 ± 0.80) as compared to control, SVP, CoQ10, and SVP + CoQ10-treated groups. CoQ10 resulted in a significant (*P* = 0.0001) increase in the percentage of positive Nissl-stained cells in CA1 (59.90 ± 5.99), CA3 (43.70 ± 1.73), and DG (98.10 ± 2.83) in contrast with rats receiving SVP in CA1 (44.00 ± 1.21), CA3 (32.80 ± 1.16), and DG (80.50 ± 1.92). The group receiving the combination of both drugs (SVP + CoQ10) showed a significant (*P* < 0.0001) increase in the percentage of positive Nissl-stained cells in CA1 (62.60 ± 1.17), CA3 (43.10 ± 1.38), and DG (98.40 ± 0.56) than in an epileptic group and SVP-treated rats. In addition, SVP + CoQ10-treated groups showed non-significant differences (*P* > 0.73, *P* > 0.99, *P* > 0.99, respectively) versus the CoQ10 group in CA1, CA3, and DG.

**Fig. 15:**
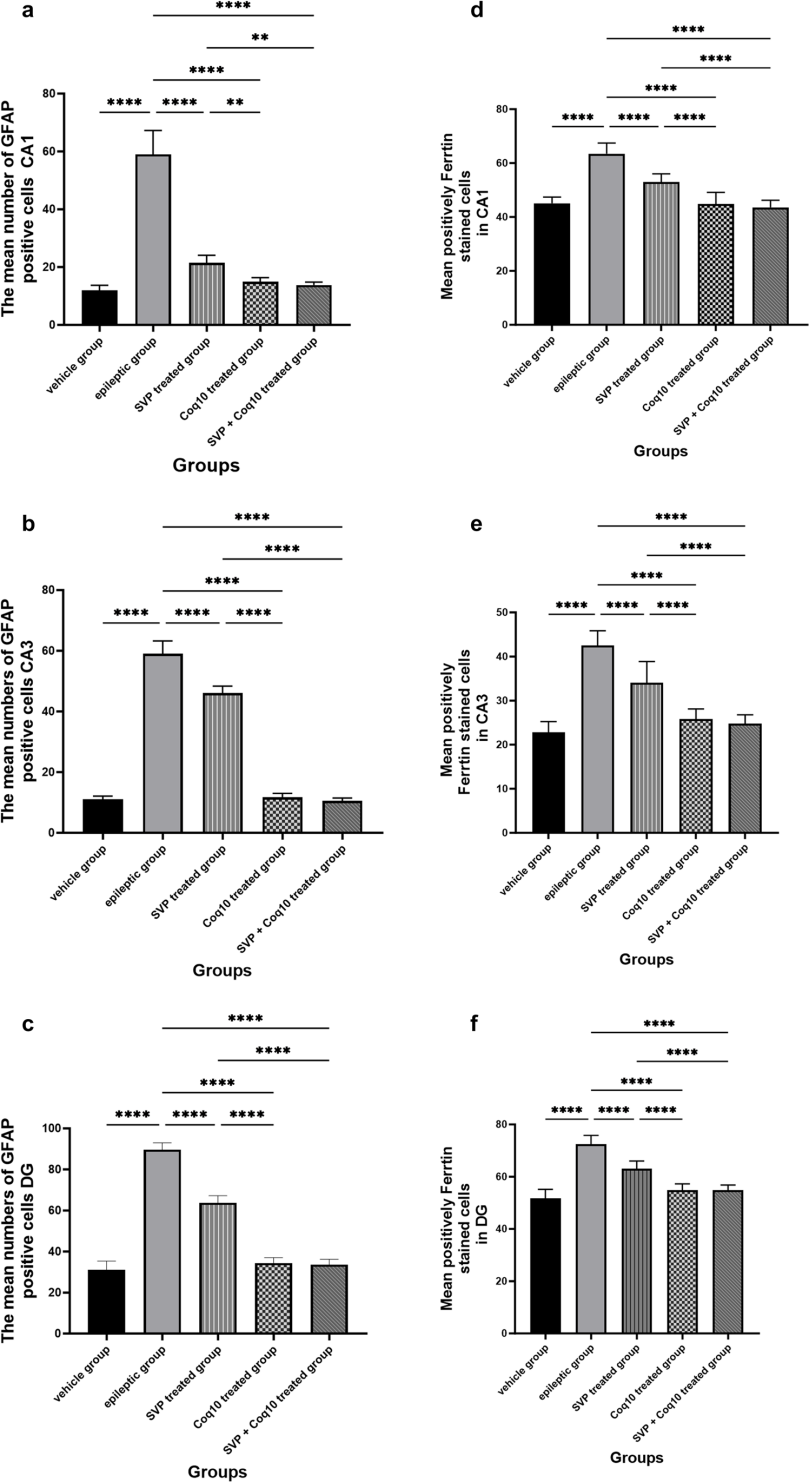
Effect of COQ10 alone or combined with sodium valproate on the morphometric hippocampus results of the hippocampus in different groups in CA1, CA3, and DG regions. The mean number of GFAP-positive cells (**a**–**c**) and the mean number of ferritin-positive cells (**d**–**f**). **Significance at *p* < 0.01 and ****significance at *p* < 0.0001

As shown in Fig. 15a–c, lithium-pilocarpine caused a significant (P < 0.0001) increase in the mean number of GFAP-positive cells in CA1 (59.00 ± 2.61), CA3 (59.00 ± 1.35), and DG (89.60 ± 1.05) as compared to control, SVP, CoQ10, and SVP + CoQ10-treated groups. CoQ10 resulted in a significant (P = 0.0001) decrease in the percentage of GFAP-positive cells in CA1 (14.90 ± 0.45), CA3 (11.70 ± 0.42), and DG (34.40 ± 0.83) in contrast with rats receiving SVP in CA1 (21.50 ± 0.81), CA3 (46.10 ± 0.70), and DG (63.70 ± 1.09). The group receiving the combination of both drugs (SVP + CoQ10) showed a significant (P < 0.0001) decrease in the percentage of GFAP-positive cells in CA1 (13.80 ± 0.32), CA3 (10.50 ± 0.30), and DG (33.60 ± 0.83) than in the epileptic group and SVP-treated rats. In addition, SVP + CoQ10-treated groups showed a non-significant difference (P > 0.99, P > 0.94, P > 0.99, respectively) versus the CoQ10 group in CA1, CA3, and DG.

As shown in Fig. 15d–f, lithium-pilocarpine caused a significant (*P* < 0.0001) increase in the mean number of FE-positive cells in CA1 (63.50 ± 1.24), CA3 (42.50 ± 1.05), and DG (72.50 ± 1.05) as compared to control, SVP, CoQ10, and SVP + CoQ10-treated groups. CoQ10 resulted in a significant (*P* < 0.0001) decrease in the percentage of FE-positive cells in CA1 (44.90 ± 1.34), CA3 (25.80 ± 0.72), and DG (54.80 ± 0.77) in contrast with rats receiving SVP in CA1 (53.00 ± 0.9545), CA3 (34.00 ± 1.53), and DG (63.00 ± 0.95). The group receiving the combination of both drugs (SVP + CoQ10) showed a significant (*P* < 0.0001, *P* < 0.0001, respectively) decrease in the percentage of FE positive cells in CA1 (43.60 ± 0.83), CA3 (24.80 ± 0.62), and DG (54.80 ± 0.62) than in an epileptic group and SVP-treated rats. In addition, SVP + CoQ10-treated groups showed a non-significant difference (*P* > 0.99) versus the CoQ10 group in CA1, CA3, and DG.

## Discussion

We found that pretreatment with COQ10 dramatically reduced seizure activity and severity during the acute phase of lithium-pilocarpine-induced seizure model, as measured by Racine’s scale. In addition, it shielded the nerve cells. Additionally, pretreatment with CoQ10 alone or in combination with SVP indicates the potential therapeutic uses for curing seizure-associated illnesses like epilepsy by targeting the ferroptosis process.

Pilo-induced seizures are ideal models for studying SE (Wu and Wang 2018). Numerous scientists and pharmaceutical companies have utilized the model to understand the causes of epilepsy better and develop more potent treatments for the disorder. Three 100 mg/kg injections were sufficient to produce continuous seizure activity, as found in the prior study (Mao et al. 2019). Evidence for ferroptosis in epileptic seizures was identified by Mao et al., who observed mitochondrial shrinkage and an increase in PTGS2 mRNA in mice that had been treated with Pilo (Mao et al. 2019). Indirect activation of T-lymphocytes and mononuclear cells by Li increases serum IL-1 levels, which in turn alters the permeability of the blood–brain barrier (BBB) and increases the uptake of Pilo. In addition, pretreatment with LiCl has been shown to boost acetylcholine release, which in turn causes more acetylcholine to cross the synaptic cleft and reach the postsynaptic membrane, where it activates muscarinic receptors and shortens the latency to initiation of SE. LiCl Pilo is a promising model for researching SE because it produces more consistent and protracted seizures with reliable results and a low death rate (Juvale and Has 2020).

Ferroptosis, a novel form of regulated cell death, was previously found in an organotypic hippocampal slice culture model of rats with glutamate-induced neurotoxicity, neurodegenerative diseases, and ischemia/reperfusion injury (Li et al. 2017; Skouta et al. 2014; Tuo et al. 2017). Ferroptosis initiation and execution lie in five critical events: iron accumulation, GPX4, GSH depletion, and lipid peroxides accumulation (Chen et al. 2020). An overview of ROS and iron’s roles as initiators and mediators of ferroptosis was presentedby Dixon and Stockwell. They discovered that iron-dependent ROS buildup occurs when the cystine-glutamate antiporter is inhibited, and glutathione levels are low. When ROS reacts with polyunsaturated fatty acids in membrane lipids, lipid peroxidation can occur (Dixon and Stockwell 2014). Ferroptosis was hypothesized to play a role in brain disorders, particularly pilocarpine-induced seizures, because of the brain’s high concentration of phospholipids and susceptibility to lipid peroxidation.

As an interesting side effect, AEDs and other drugs also influence molecules involved in the ferroptosis process. For example, lipid peroxidation levels were dramatically increased in epileptic youngsters using therapeutic doses of levetiracetam (Haznedar et al. 2019). In addition, the adverse effects of SVP treatment in patients (CENGIZ et al. 2000) and animal models (Tong et al. 2005) are associated with OS and reductions in antioxidant enzymes, including GPx, SOD, and catalase.

CoQ10 is an effective natural antioxidant with a fundamental role in cellular bioenergetics and numerous known health benefits. By regulating ROS generation and controlling cellular redox status, it protects cells from damage caused by free radicals. CoQ10 is capable of suppressing inflammatory gene expression and so has antiinflammatory properties. Additionally, it may aid in immunity by controlling lysosomal and peroxisomal activity throughout the immune response (Zhao et al. 2022). Recent clinical trials and experimental research have documented that CoQ10 consumption provides remarkable protection against acute organ injuries (including but not limited to brain, heart, lung, liver, and kidney damage) (Ali et al. 2021; Chen et al. 2021; Li et al. 2020; Zhao et al. 2022). CoQ10 is a powerful antioxidant that provides neuroprotection in some forms of neurodegenerative illness (Mancuso et al. 2010). CoQ10 can penetrate the BBB and accumulate neuroprotective amounts in the brain. CoQ10 may provide more protection to neurons than common antioxidants like vitamin E (Abdin and Hamouda 2008; Aboul-Fotouh 2013). This suggests that CoQ10’s ability to scavenge free radicals may contribute slightly to its positive effect. CoQ10 provides neuroprotection in animal models of neurodegenerative disorders, including Alzheimer’s and Parkinson’s, whose pathophysiology is linked to mitochondrial dysfunction (Abdin and Hamouda 2008; Aboul-Fotouh 2013; Yang et al. 2009). In epilepsy, lipid peroxidation is accompanied by a reduction in CoQ10 that aggravates the condition, and exogenous CoQ10 administration could reverse this scenario (Tawfik 2011).

Interestingly, no study detected the targeted effect of COQ10 on hippocampal ferroptosis in a SE rat model. However, there was a different study on the neuroprotective effect of COQ10 in epileptic models in rodents (Ahras-sifi and Laraba-djebari 2020; Bhardwaj and Kumar 2016; Mao et al. 2019).

CoQ10 pretreatment significantly reduced seizure activity and severity as measured by Racine’s scale in the current lithium-pilocarpine paradigm. Tawfik found that the effects of pilocarpine-induced seizures could be mitigated by taking coenzyme Q10 (CoQ10). It boosted the effectiveness of phenytoin as an antiepileptic medication. CoQ10 was also a useful adjunct to phenytoin treatment for patients with pilocarpine-induced seizures (Tawfik 2011). Researchers Baluchnejadmojarad et al. concluded that pretreatment with CoQ10 could reduce the severity of spontaneous recurrent seizures and prevent the death of hippocampal neurons and the growth of abnormal mossy fibers in the DG’s inner molecular layer following kainate administration in a rat model (Baluchnejadmojarad and Roghani 2013).

Consistent with prior research utilizing the pilocarpine model, our results showed an increase in MDA and a depletion of the antioxidant enzyme pool, as revealed by decreases in GSH in hippocampus homogenates (Ali et al. 2018; Cao et al. 2021; Tawfik 2011). The free radical damage caused by OS and the excitotoxicity caused by an overabundance of neurotransmitters may explain all of this (Santos et al. 2009). Epileptic rat brain homogenates treated with CoQ10 during the acute phase of pilocarpine-induced convulsions revealed decreased lipid peroxidation, corroborating the findings of prior studies on the influence of antioxidants on pilocarpine-induced oxidative stress and neuronal damage (Diniz et al. 2015; Pearson and Patel 2016; Shakeel et al. 2017). They also improved antioxidant indexes, which indicated a decreased risk of OS. Pilo-induced seizures were also prevented, and their intensity was reduced when the animals were pretreated with CoQ10. This was likely due to a reduction in the OS in the rat hippocampus that resulted from the seizures. An increase in antioxidant enzyme activities, while decreasing free radical generation, significantly reduces vulnerability to pilocarpine-induced seizures, as emphasized by Santos et al. (2009). CoQ10 enhanced the antioxidant and antiepileptic benefits of SVP treatment, indicating that CoQ10 may be used as an adjuvant to standard AEDs. Treatment with SVP has been linked in some research to changes in iron metabolism in epilepsy, with results including increased OS and the development of non-transferrin-bound iron. The serum iron, ferritin, and transferrin saturation levels were all within normal ranges in epileptic patients (Ounjaijean et al. 2011).

Ferritin is most cells’ primary intracellular iron-storage protein (Cheli et al. 2020). The current investigation utilized FE as a marker of altered iron metabolism. Observable alterations to this protein indicate potentially abnormal iron metabolism. This study demonstrated that epileptic rats have a significantly higher FE level than control rats. Coenzyme Q10 also reduced FE protein levels. Additionally, the benefits of SVP therapy for regulating iron stores were amplified by incorporating CoQ10. In tubular sclerosis tissues, an important contributor to treatment resistance in epilepsy, increased ferritin expression and intracellular iron accumulation have been observed (Zhou et al. 2022).

Since GPX4 is a selenium-dependent enzyme for interneuron growth and seizure prevention, these findings provide evidence for the crucial role of GPX4 in epilepsy (Ingold et al. 2018). These findings show that GPX4 protein expression is downregulated in a SE rat model. CoQ10 also raised the GPX4 protein concentration. Also, GPX4 was downregulated in kainic acid-treated rats, and ferrostatin-1 (Fer-1) was able to correct this (Ye et al. 2019). In addition, PTZ and Pilo-treated mice had lower levels of GPX4 protein, which Fer-1 reversed (Mao et al. 2019). The negative correlation between the levels of GPx4 and lipid peroxidation marker and FE level suggests that the decrease in GPX4 activity may account for the intensity of lipid peroxidation and FE level. In contrast, a significant positive correlation was found between GPx4 activity and GSH intensity. This could be explained by Cappelletti et al. (2020) and Forcina and Dixon (2019). The protein GPX4 prevents lipid peroxidation in cell membranes. But GPX4 can turn lipid hydroperoxides into lipid alcohols with the help of GSH (Capelletti et al. 2020).

Histological and immunohistochemical evaluations were carried out to confirm the biochemical findings. Degenerated and disordered pyramidal cells were seen in regions CA1 and CA3 of the hippocampus proper in the epileptic group, as revealed by this study’s light microscopic analysis. Most pyramidal cells showed degenerative changes, and vacuoles surrounded their elongated, deeply stained nuclei. A change in the arrangement of pyramidal cells could constitute an adaptive response to pressure. This could be the pyramidal cells’ first attempt at recovering their former abilities. Consistent with these results, additional researchers demonstrated a highly disordered hippocampal pyramidal cell layer (Tsegay et al. 2021). Degenerative alterations of nerve cells with vacuolated pericellular space were also observed in DG from the epileptic group. Vacuolated pericellular hallows developed primarily because nerve cells shrank and withdrew their cytoplasmic processes because of the dissolution of their cytoskeletal elements. It was established that oxidative stress, which causes free radicals to assault neural cells, can lead to nerve cell degeneration (Wu and Wang 2018). They further speculated that endothelial damage, vasodilatation, and increased vascular permeability might all play a role in creating the interstitial edema that leads to wide interstitial spaces between pyramidal cell neurons (Wu and Wang 2018).

Dispersion of neurons in CA1, CA3, and DG of the hippocampus was also seen with Nissl staining after pilocarpine administration. The previous explanation was the absence of Reelin, a protein crucial for neuronal migration and lamination (Cooper 2008). There can be no protein depletion without Nissl bodies doing their crucial role. A decrease in Nissl-stained cells shows the suppression of neuronal protein production. Evidence like this points to pilocarpine’s deleterious effects on neuronal function (Parent et al. 1999). On the other hand, the hippocampi of pilocarpine-exposed rats showed compacted Nissl granules in the cytoplasm of pyramidal cells and occasional darkly stained basophil neurons (Liu et al. 2003).

CoQ10 pretreatment has been shown to enhance the number of cells that stain positively for Nissl stain, which is an interesting finding. CoQ10’s considerable neuroprotection characteristics have been demonstrated, suggesting that it may shield neurons from damage in the event of neurodegenerative disease (Tawfik 2011). CoQ10 pretreatment in a spinal cord contusion model enhanced neurological functioning and normal motor neuron survival (Hwang et al. 2015).

Microglia are cells that clean tissue by phagocytosis after generating proinflammatory cytokines to combat invading agents and harmful substances in the brain. Ferritin is a protein that is produced by microglia. There is mounting evidence that ferritin is a key player in inflammation (de Rodríguez‐Callejas et al. 2019). Our research shows that microglial cells in the hippocampus of epileptic rats exhibit elevated ferritin immunoreactivity compared to control expression. The most common form of cell that expresses ferritin resembles microglia in appearance, with a tiny, irregular cell body and branching processes. Another kind of cell that tested positive for ferritin had a tiny, spherical soma and no processes. Oligodendrocytes were likely the less numerous cells in this group than microglia cells (de Rodríguez‐Callejas et al. 2019). Treatment with CoQ10 alone or in combination with SVP reduced ferritin expression in epileptic rats. Gorter et al. also reported a similar finding (Gorter et al. 2005). They discovered that iron could bring on seizures. Whether this effect is due to iron-induced lesions or iron-induced destabilization of neighboring neural networks is unknown. CoQ10 is a lipophilic radical scavenger in the membrane and a lipophilic electron transporter in the mitochondrial respiratory chain (Santoro 2020). The antioxidant benefits of non-mitochondrial CoQ10, of which ferroptosis suppressor protein 1 is an important component, were discovered by Bersuker et al. to be exerted by the recruitment of this protein into the plasma membrane(Bersuker et al. 2019). CoQ10 and GSH were found to have an antiferroptosis impact in another investigation (Shimada et al. 2016).

Many CNS cells, including astrocytes, contain GFAP, an intermediate filament protein. GFAP plays a significant role in regulating astrocyte motility and form by providing structural stability to astrocytic processes. Finally, GFAP is a mature CNS astrocyte-specific marker (Moeton et al. 2016). Astrogliosis was discovered to result from CNS injury caused by trauma, disease, genetic diseases, or chemical factors. Astrogliosis is characterized by increased GFAP production, which can be observed through immunostaining with an antibody against GFAP (Yuan et al. 2021). The present study measured pilocarpine-induced astrocyte activation by dramatically increasing GFAP expression. There is a possibility that this is a protective response to pilocarpine-induced neuronal injury. In other types of neurotoxicity, these alterations have been demonstrated before (Mirrione et al. 2010; Vargas-Sánchez et al. 2018). Faint GFAP immunoreaction was observed in CoQ10 with or without SVP, suggesting that therapy with CoQ10 alone or in combination with SVP positively influences the affected astrocytes in the CA1 and CA3 areas and in the DG of the hippocampus. According to Borowicz-Reutt et al., this discovery can be explained by fewer astrocytes because the neuronal structure is slowly regaining its normal state. Activation of astrocytes involves neurotoxic activities generated by oxidative insults and is a key component of the brain’s antioxidant defense mechanism (Borowicz-Reutt and Czuczwar 2020).

## Conclusion

Our results imply that CoQ10 has been promised as a safe and effective complement to SVP therapy in epilepsy since it decreases seizure severity and protects against seizure-induced oxidative damage and ferroptosis-related damage in pilocarpine-treated rats. In addition, our results provided compelling evidence that CoQ10 could be a useful adjuvant for protecting against oxidative damage and ferroptosis-related damage that result from epileptic seizures.

## Data Availability

The data used to support the findings of this study are included in the article.
